# Quantifying the metabolic capabilities of engineered *Zymomonas mobilis *using linear programming analysis

**DOI:** 10.1186/1475-2859-6-8

**Published:** 2007-03-09

**Authors:** Ivi C Tsantili, M Nazmul Karim, Maria I Klapa

**Affiliations:** 1 Metabolic Engineering and Systems Biology Laboratory, Institute of Chemical Engineering and High-Temperature Chemical Processes, Foundation for Research and Technology-Hellas, GR-26504, Patras, Greece; 2 Interdepartmental Graduate Program "Mathematical Modelling in Modern Technologies and Finance", National Technical University of Athens, Zografou Campus, GR-15780, Athens, Greece; 3 Department of Chemical Engineering, Texas Tech University, Lubbock, TX 79409, USA; 4 Department of Chemical and Biomolecular Engineering, University of Maryland, College Park, MD 20742, USA; 5 Present address: Department of Naval Architecture and Marine Engineering, National Technical University of Athens, Zografou Campus, GR-15780, Athens, Greece

## Abstract

**Background:**

The need for discovery of alternative, renewable, environmentally friendly energy sources and the development of cost-efficient, "clean" methods for their conversion into higher fuels becomes imperative. Ethanol, whose significance as fuel has dramatically increased in the last decade, can be produced from hexoses and pentoses through microbial fermentation. Importantly, plant biomass, if appropriately and effectively decomposed, is a potential inexpensive and highly renewable source of the hexose and pentose mixture. Recently, the engineered (to also catabolize pentoses) anaerobic bacterium *Zymomonas mobilis *has been widely discussed among the most promising microorganisms for the microbial production of ethanol fuel. However, *Z. mobilis *genome having been fully sequenced in 2005, there is still a small number of published studies of its *in vivo *physiology and limited use of the metabolic engineering experimental and computational toolboxes to understand its metabolic pathway interconnectivity and regulation towards the optimization of its hexose and pentose fermentation into ethanol.

**Results:**

In this paper, we reconstructed the metabolic network of the engineered *Z. mobilis *to a level that it could be modelled using the metabolic engineering methodologies. We then used linear programming (LP) analysis and identified the *Z. mobilis *metabolic boundaries with respect to various biological objectives, these boundaries being determined only by *Z. mobilis *network's stoichiometric connectivity. This study revealed the essential for bacterial growth reactions and elucidated the association between the metabolic pathways, especially regarding main product and byproduct formation. More specifically, the study indicated that ethanol and biomass production depend directly on anaerobic respiration stoichiometry and activity. Thus, enhanced understanding and improved means for analyzing anaerobic respiration and redox potential *in vivo *are needed to yield further conclusions for potential genetic targets that may lead to optimized *Z. mobilis *strains.

**Conclusion:**

Applying LP to study the *Z. mobilis *physiology enabled the identification of the main factors influencing the accomplishment of certain biological objectives due to metabolic network connectivity only. This first-level metabolic analysis model forms the basis for the incorporation of more complex regulatory mechanisms and the formation of more realistic models for the accurate simulation of the *in vivo Z. mobilis *physiology.

## Background

In the highly energy-consuming and earth-polluting era of the early 21^st ^century, the need for discovery of alternative, renewable, environmentally friendly energy sources and the development of cost-efficient, environmentally clean methods for their conversion into higher fuels becomes more than imperative. Ethanol's significance as fuel has dramatically increased in the last decade [1] due to characteristics that render it more effective than gasoline in optimized engines [2], with the additional advantage of contributing less to the green house effect than the conventional fuel. Ethanol, among other effective fuels, could be produced from hexoses and pentoses through microbial fermentation [3-8]. Importantly, plant biomass, which constitutes one of the main renewable energy sources on earth, could provide a significant and inexpensive source of the hexose and pentose mixture, if appropriately and effectively depolymerized [2,9-11],. In this context, optimization of the hexose and pentose microbial fermentation into ethanol is of great importance. Metabolic engineering (ME) can significantly contribute towards this end with its experimental and computational toolboxes [12-14].

To-date, *Saccharomyces cerevisiae *[15-17] and *Escherichia coli *[18-20] have been the main industrial microorganisms utilized for ethanol production, with *Klebsiella oxytopa*, *Pichia stipitis and pastoris *[2,19] being studied as potential candidates. Recently, the anaerobic *Zymomonas mobilis *is being also discussed among the most promising microorganisms for the microbial conversion of hexoses and pentoses into ethanol fuel due to numerous advantageous characteristics [17]. Its ethanol yield reaches 98% of the theoretical maximum compared to ~90% of *S. cerevisiae *[17]. *Z. mobilis *is the only to-date identified bacterium that is toxicologically tolerant to high ethanol concentrations [2,21], requiring thus less intricate and consequently less expensive downstream processing for the removal of ethanol in industrial chemical plants. Moreover, it has (i) low biomass yield [22], biomass competing with ethanol for the available carbon source(s), (ii) high speed of substrate conversion to metabolic products [17], and (iii) comparatively simple glycolytic pathways [21], fact that might prove beneficial for this organism's cell engineering towards the optimization of the ethanol production process. In addition, any disadvantages of the *Z. mobilis *use for ethanol production in the food and beverage industry, referring mainly to the formation of byproducts modifying food flavor [17], are not applicable in the context of biofuel production. Finally, its wild-type not catabolizing pentose sugars, *Z. mobilis *engineering [24] resolved the last major obstacle associated with its use for the fermentation of plant biomass [25].

Despite, however, the increasing interest in *Z. mobilis*, the number of reports in current literature studying its *in vivo *physiology remains small [22,23,26-29]. This implies a rather limited so far use of the metabolic engineering toolbox for the analysis of the microorganism's metabolic pathway interconnectivity and regulation. The recent publication of *Z. mobilis *full genome [21] is expected to greatly assist the investigations for the identification of potential genetic modification targets towards optimized *Z. mobilis *strains. In this context, the main objectives of the presented work, discussed sequentially in the following sections, were (a) to reconstruct the metabolic network of the engineered *Z. mobilis *using the available resources to a level that it could be modeled according to the existing metabolic engineering methodologies, and (b) to use linear programming (LP) analysis – the first level of metabolic modeling towards the simulation of *in vivo *physiology [30-37] – for the identification of the microorganism's metabolic boundaries with respect to various biological objectives, as these boundaries are determined only by the stoichiometric connectivity of the network.

## Results and Discussion

### A. Reconstruction of the *Z. mobilis *Metabolic Network

The reconstruction of an organism's metabolic network used to be mainly based on the existing knowledge about the metabolic network structure of similar cellular systems, along with any available data regarding *in vitro/in vivo *enzymatic activity and metabolic output measurements under various genetic backgrounds or environmental conditions [see e.g. [38]]. In the post-genomic era, the available resources are further augmented by the ever-increasing knowledge about gene annotation based on high-throughput sequencing [e.g. [33,39,40]] and gene expression analyses [41]. While the availability of the genomic data provides a significant advancement in the process of reconstructing the maximum potentially active metabolic network of a biological system, this remains a non-trivial task that requires the direct involvement of an expert's judgment to decide over the sometimes multiple feasible answers to questions that arise during the process [42].

**Figure 1:**
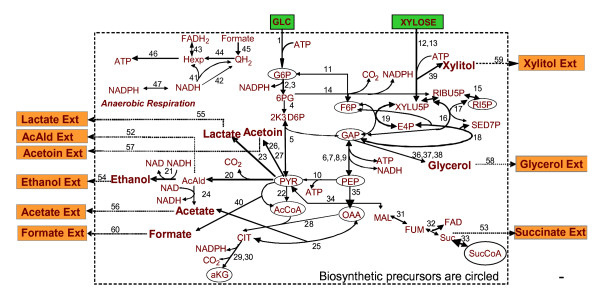
The *Z. mobilis *reconstructed metabolic network. The numbers next to the reaction arrows refer to the reaction listing in Appendix 1A. Biomass precursors are circled.

The reconstructed metabolic network of the engineered *Z. mobilis *is depicted schematically in Figure 1, while all included reactions are listed in Appendix 1A (in the rest of the text all reactions will be referred by their number in Appendix 1A). The main utilized resources were the available annotation of the recently fully sequenced *Z. mobilis *genome [21], the public metabolic databases KEGG [43] and EXPASY [44], the *Z. mobilis in vivo *flux analysis studies [22,23] and biochemistry textbooks [45,46]. Specifically, *Z. mobilis *utilizes the Entner-Doudoroff (E.D.) and part of the Embden-Meyerhof-Parnas pathway (E.M.P) for the catabolism of glucose into pyruvate (reactions 1–11) that leads to the production of 1 mole of ATP, NADPH and NADH. Xylose isomerase, xylulokinase and the full pentose phosphate pathway (PPP) (reactions 12–19) were considered as potentially active to account for the catabolism of pentoses by the engineered strain. Because xylitol has been observed as product of an engineered *Z. mobilis *strain under a particular set of conditions [22], reaction 39 was also included in the reconstructed network.

No α-ketoglutarate dehydrogenase gene has yet been annotated in the *Z. mobilis *genome, supporting the current hypothesis that the anaerobic *Z. mobilis *features an incomplete citric acid cycle (TCA) (reactions 28–33) [22,23]. This is in agreement with prior biological knowledge [45], according to which fermenting organisms transform TCA from an oxidative to a reductive pathway. In this case, the two separate parts of the TCA cycle serve in producing the biosynthetic precursors α-ketoglutarate (left branch in Figure 1), oxaloacetate and succinyl-CoA (right branch in Figure 1). The "right" (in figure 1) branch is also connected to the anaerobic respiration, since under anaerobic conditions fumarate could act as electron acceptor and be reduced to succinate through a membrane-bound fumarate reductase enzyme (reaction 32) [45]. According to the currently available genomic and metabolic information, the *Z. mobilis *oxaloacetate pool is replenished by two anaplerotic reactions catalyzed by the enzymes phosphoenolpyruvate carboxylase (reaction 35) and malate dehydrogenase (reaction 34). Anaerobically growing *Z. mobilis *can feature a number of fermentation reactions (reactions 20–27). These reactions and their connection with anaerobic respiration are currently considered the determining factor for the *Z. mobilis *ability to produce ethanol in high yields [17].

Major role in a metabolic network's reconstruction and further modeling plays the selection of the respiration reactions (reactions 41–47). While no clear indication of the activity of the formate dehydrogenase (reaction 40) complex with ubiquinol-cytochrome c reductase (reaction 45) currently exists, reaction 45 was still included in the stoichiometric model [36], formate considered among the potential products of the anaerobic microorganism. An additional assumption, which is not currently backed up by genomic information, is the activity of NAD(P) transhydrogenase (it will be referred as trans in the rest of the text) (reaction 47); including this reaction in the stoichiometric model, NADH and NADPH become equivalent [35]. Finally, the considered amino acid biosynthesis and cumulative biomass formation reactions (reactions 61–79) were based on the information of Table 1, the latter being populated after appropriately modifying Table 2 in [22]. Among the modifications, methionine biosynthesis, which in [22] was considered as catalyzed by the EC 2.3.1.46 enzyme, was replaced by reaction 71 catalyzed by the EC 2.3.1.31 enzyme, the latter being identified as potentially active in *Z. mobilis *[43]; according to current knowledge, only one of the two enzymes could be potentially active in an organism, never both [47].

**Table 1:** *Z. mobilis *biosynthetic requirements

		**Precursor Stoichiometry (mol/mol amino acid)**															
		
Amino acid	Amount (μmol/g dry weight)	**G6P**	**F6P**	**RI5P**	**E4P**	**GAP**	**G3P**	**PEP**	**PYR**	**AcCoA**	**OAA**	**AKG**	**CO2**	**NADPH**	**NADH**	**Succ**	**Suc CoA**
Ala	1088								1		1		-1	1			
Arg	181											1	1	4			
Asx	478										1			1			
Cys	20						1							5	-1		
Glx	343											1		1			
Gly	920						1							1	-1		
His	82			1										1	-2		
Ile	369								1		1		-1	5			
Leu	369								2	1			-2	2	-1		
Lys	249								1		1		-1	4		-1	1
Met	81										1			8	-1		
Phe	11				1			2					-1	2			
Pro	210											1		3			
Ser	202						1							1	-1		
Thr	224										1			3			
Trp	54			1	1			1					-1	2			
Tyr	70				1			2					-1	2			
Val	569								2				-1	2			

Precursor Amount (μmol/g dry weight)																	

Polymer	**G6P**	**F6P**	**RI5P**	**E4P**	**GAP**	**G3P**	**PEP**	**PYR**	**AcCoA**	**OAA**	**AKG**	**CO2**	**NADPH**	**NADH**	**Succ**	**Suc CoA**	

RNA			600			350				250			1550	-406			
DNA			87			44				44			174	-65			
Lipids					120	120			2048				3615	-120			
Peptidoglycam		190					95	285	190	95	95		760				
Glycogen	154																
C1-Units						49							49	-49			
Polyamines											59		180				

**Table 2:** Maximization of Biosynthetic Precursor Production for glucose or xylose substrate cases.

		**G6P (6)**	**F6P (6)**	**RI5P (5)**	**E4P (4)**	**GAP (3)**	**G3P (3)**	**PEP (3)**	**PYR (3)**	**AcCOA (2)**	**OAA (4)**	**AKG (5)**	**SucCoA (4)**
***Glc***	**Yield**	0.921	0.921	1	1	1	1	1	1.3	1.3	1	0.6	1.645
	**C Conversion**	92.1%	92.1%	83.3%	66.67%	50%	50%	50%	65%	43.3%	66.67%	50%	110%
	**ATP dual price**	-0.079	-0.079	0	0	0	0	0	0.3	0.3	0	0.2	-0.097
	**Constrained by**	Energy	Energy	Stoich	Stoich	Stoich	Stoich	Stoich	Energy	Energy +Stoich	Stoich	Energy+ Stoich	None

***Xylose***	**Yield**	0.667	0.667	0.9	1	1	1	1	1.033	1.033	1	0.511	1.387
	**C Conversion**	80%	80%	90%	80%	60%	60%	60%	62%	41.3%	80%	51.1%	111%
	**ATP dual price**	0	0	-0.1	0	0	0	0	0.3	0.3	0	0.2	-0.097
	**Constrained by**	Stoich	Stoich	Energy	Stoich	Stoich	Stoich	Stoihc	Energy	Energy +Stoich	Stoich	Energy+Stoich	None

The table depicts the maximum production rate of each precursor (yield), the C conversion, the ATP dual price, and the factor (energy or stoichiometry or both) that constrains higher precursor yield (see Materials and Methods). In parenthesis under the abbreviation of each precursor, the number of its carbon atoms is depicted.

In summary, the reconstructed metabolic network comprises 79 reactions and 77 metabolites, among which 19 and 18, respectively, participate solely in the amino acid biosynthesis and cumulative biomass formation reactions. The potential reversibility of the reactions was determined based on currently available knowledge provided mainly from metabolic databases [43,44]. Among the metabolites, two (glucose Ext-M47 and xylose Ext-M48) were considered as potential substrates, while nine were considered as potential products (Acetaldehyde (AcAld) Ext-M51, Succinate (Suc) Ext-M52, Ethanol Ext-M53, Lactate Ext-M54, Acetate Ext-M55, Acetoin Ext-M56, Glycerol Ext-M57, Xylitol Ext-M58, Formate Ext-M59), based primarily on data from *in vivo *experiments [22] (the number of each metabolite refers to its listing in Appendix 1B). In the rest of the text, all metabolites will be referred by their abbreviations shown in Appendix 1B.

### B. Linear Programming Analysis

#### B1. Production of Cofactors

**Figure 2:**
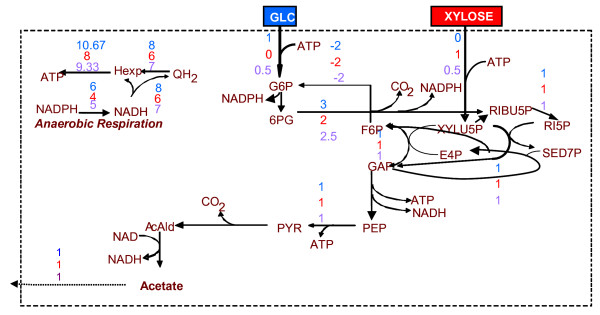
Optimal flux distribution for maximization of the ATP production rate. The flux values next to each reaction arrow refer to the three examined substrate cases (from upper to lower): (a) glucose as single substrate, (b) xylose as single substrate, and (c) 50% of each. Due to the linearity of problem, the solution of the latter case is an interpolation of the first two.

The maximum ATP production (11.667 moles ATP per mole glucose, 9 moles ATP per mole xylose and 10.333 moles ATP per 0.5 mole glucose and 0.5 mole xylose) requires the use of the PPP leading to the production of acetate (see Figure 2). This route is preferred, because it allows for the largest possible, under the particular circumstances, NADH and NADPH production. Specifically, based on the stoichiometry of the respiration reactions, production of 1 mole NADH or NADPH corresponds to production of 1.333 mole ATP. This explains the -1.333 dual price of NADPH or NADH in all three examined substrate cases.

In the case that the ATP production rate is imposed to be equal to the ATP consumption rate, maximum NADH or NADPH production (6.5 moles of NADH or NADPH per mole glucose, 4.5 moles NADH or NADPH per mole xylose, 5.5 moles NADH or NADPH per 0.5 mole xylose and 0.5 mole glucose) requires the use of the PPP leading to the production of both acetate and glycerol. Glycerol production, although competing with the objective as it consumes NADH, is part of the solution, because of the "ATP balance equal to zero" constraint and the fact that the stoichiometry of the network provides limited number of metabolic routes for the consumption of ATP. Specifically, the stoichiometry of the network after the GAP node favours only the production of ATP, providing no options for the consumption of the produced ATP. This explanation for the glycerol production is supported by the dual price of ATP, which is equal to 1.5 in all three examined substrate cases. This indicates that the maximum NADH or NADPH production would have increased by 1.5 mole, should the capability of the network to consume ATP have increased by 1 mole. This is indeed the case when no constraint is imposed on the ATP balance. Then, the substrate(s) is(are) fully converted to acetate, while the maximum production of NADH or NADPH is increased by 1.5 mole.

#### B2. Production of Biosynthetic Precursors

*Z. mobilis *has 12 biosynthetic precursors, namely the elements G6P, F6P, RI5P, E4P, GAP, G3P, PEP, PYR, AcCoA, OAA, AKG and succinyl-CoA (see Appendix 1B). *Z. mobilis *capability to produce each one of those is shown in Table 2 (see Materials and Methods for the formulation of the LP problem). Interestingly, no full conversion of glucose or xylose to G6P/F6P or RI5P, respectively, is observed, despite the latter being the immediate product(s) of the former. The microorganism's capability to produce G6P/F6P or RI5P from glucose or xylose, respectively, is limited by energy requirements. This is true, because conversion of 1 mole of glucose to 1 mole of G6P/F6P or 1 mole of xylose to 1 mole of RI5P is accompanied by the consumption of 1 mole ATP. Due to the "ATP balance should be equal to zero" constraint, this ATP consumption imposes the activation of ATP producing routes in addition to the catabolic reaction "Glc → G6P/F6P" or "Xylulose → RI5P". The "equal to zero" constraint on the ATP balance explains also the fact that the PYR (and consequently AcCoA) production is constrained by energy requirements, while this is not the case for the immediate precursors of pyruvate (PYR), GAP and G3P. Finally, the production of AKG, which is accompanied by NADP reduction, is also constrained by energy requirements in both glucose and xylose substrate cases. This is indicated by the ATP and NADPH positive dual prices, when considered that NADPH can be transformed to ATP through the anaerobic respiration.

#### B3. Ethanol Production

**Figure 3:**
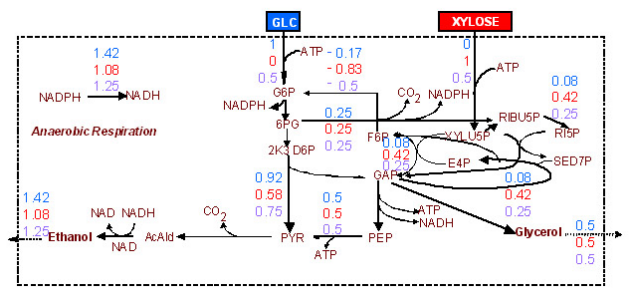
Optimal flux distribution for maximization of the ethanol production rate. No biosynthetic requirements have been considered. Depicted flux values are defined as in Fig. 2.

The maximum ethanol yield (1.42 mole ethanol per mole glucose, 1.08 mole ethanol per mole xylose and 1.25 mole ethanol per 0.5 mole glucose and 0.5 mole xylose) is connected to the catabolism of the substrate(s) through the E.D., P.P., and E.M.P pathways, while the optimal flux network involves also the production of glycerol (see figure 3). Sensitivity analysis indicated that the ethanol yield is constrained by the fact that the network lacks flexibility in consuming the ATP surplus. This is verified by the dual price of ATP, which was positive (+0.583) in all three examined substrate cases, along with the production of glycerol, despite the latter being competitive to the desired objective, as it was explained in section B1. Sensitivity analysis also showed that 0.5 mole increase in the available NADH or NADPH could lead to increase in the maximum ethanol yield equal to 0.1667 × 0.5 = 0.0833 mole, NADPH dual price being equal to -0.1667 with allowable decrease equal to 0.5 mole. Indeed, the maximum ethanol yield increased to 1.5 mole per mole glucose, 1.167 mole per mole xylose and 1.333 mole per 0.5 mole glucose and 0.5 mole xylose, when the NADH consumption rate was allowed to be smaller than its production rate. In these LP problems, the ATP dual price remains positive (+0.5), with allowable increase equal to 1 mole per mole of substrate feed, while the optimal flux distribution still comprises the production of glycerol.

If no constraint is imposed on the ATP balance, the ethanol yield is equal to the chemically allowed maximum (namely 2 moles per mole glucose, 1.667 mole per mole xylose and 1.833 mole per 0.5 mole glucose and 0.5 mole xylose). The substrate(s) is(are) fully converted to ethanol without the formation of byproducts. The result is identical in the case that the "ATP balance is equal to zero" constraint is active but the respiration reactions are considered as potentially reversible. This case is equivalent to no constraint on the ATP balance as the surplus of the produced ATP is allowed to be transformed to NADH through the anaerobic respiration.

#### B4. Biomass Production

**Figure 4:**
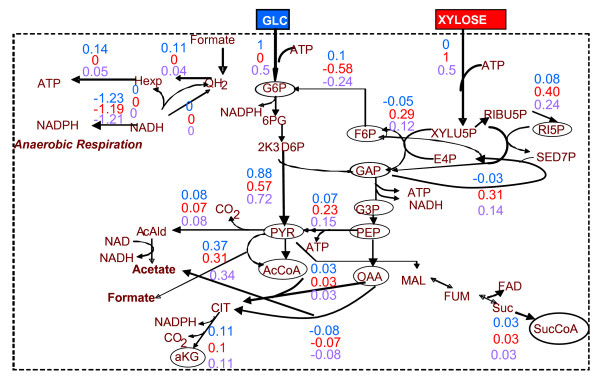
One of the optimal flux distributions corresponding to the maximum growth rate. In the case of glucose substrate, an identified alternative solution involved r40 = depicted value - 0.31 and r22 = depicted value + 0.31; this and the depicted in the figure as active route are equivalent with respect to AcCoA synthesis, thus leading to the same biomass production rate. Depicted flux values are defined as in Figures 2-3.

The maximum growth of *Z. mobilis *was estimated equal to: a) 129 g biomass per mole of glucose, b) 107.5 g biomass per mole xylose and c) 118.25 g biomass per 0.5 mole xylose and 0.5 mole glucose(see figure 4). The dual prices of all biosynthetic precursors and NADH/NADPH are shown in Table 3 for the substrate cases of glucose and xylose. It can be observed that despite their coefficients in the biomass equation being relatively small, the dual prices of G6P/F6P or RI5P for glucose or xylose as susbtrate, respectively, are the largest in absolute value. This holds true, because these biosynthetic precursors are the immediate product of the corresponding substrate. Thus, despite their small direct contribution to biomass formation, they are precursors of all the other biosynthetic molecules. The scaled dual prices in Table 3 further support this explanation, providing also information about other major biosynthetic precursors. Specifically, they indicate that when the network achieves maximum growth, the production of G6P/F6P, RI5P and Suc-CoA (in decreasing order of scaled dual prices), when glucose is the only substrate, or RI5P, Suc-CoA, G6P/F6P and E4P (the latter two have same scaled dual prices), when xylose is the only substrate, approach their maximum yield (see section B.2).

**Table 3:** Biosynthetic precursor scaled dual prices for glucose or xylose as substrates.

		**G6P**	**F6P**	**RI5P**	**E4P**	**GAP**	**G3P**	**PEP**	**PYR**	**AcCOA**	**OAA**	**AKG**	**SucCoA**	**NADH**	**NADPH**
	× **10^-4 ^moles for 1 g biomass**	1.54	1.9	8.23	1.35	1.2	17.05	3.11	38.67	26.07	28.78	8.88	2.49	-25.2	172.02

**Glc**	**Dual Price**	-129	-129	-107.5	-86	-64.5	-64.5	-64.5	-64.5	-64.5	-64.5	-129	-64.5	0	0
	**Metabolite's yield**	0.92	0.92	1	1	1	1	1	1.3	1.3	1	0.6	1.645	6.5	6.5
	**Scaled dual price σ**	-0.92	-0.92	-0.83	-0.67	-0.5	-0.5	-0.5	-0.65	-0.65	-0.5	-0.6	-0.82	0	0

***Xylose***	**Dual Price**	-129	-129	-107.5	-86	-64.5	-64.5	-64.5	-64.5	-64.5	-64.5	-129	-64.5	0	0
	**Metabolite's yield**	0.67	0.67	0.9	1	1	1	1	1.03	1.03	1	0.51	1.39	4.5	4.5
	**Scaled dual price σ**	-0.8	-0.8	-0.9	-0.8	-0.6	-0.6	-0.6	-0.62	-0.62	-0.6	-0.61	-0.83	0	0

The Table depicts also the stoichiometric coefficient of each precursor in the biomass equation, its dual price in the maximizing the growth rate solution and its maximum yield (see Table 2).

#### B.5 Ethanol production under specific biosynthetic requirements

Constraining growth to [0.1 × *n*]-fold (n ∈ N,1 ≤ *n *≤ 10) of the maximum biomass yield calculated in section B4, the maximum ethanol yield becomes equal to (1-0.1 × *n*)-fold of the maximum chemically allowed ethanol yield (see section B3) for any of the three considered substrate cases. For all values of *n*, the substrate(s) is(are) catabolized through the E.D., P.P. and E.M.P pathways, while the respiration uses the transhydrogenase (trans) reaction (r-47). For the particular biosynthetic requirements and LP constraints, the ATP and NADH/NADPH dual prices are equal to zero and the estimated maximum ethanol yield is constrained only by the stoichiometry of the network.

#### B.6. Effect of single and double gene deletions on *Z. mobilis *metabolic capabilities

Deletion of a gene in the context of the metabolic LP problem is equivalent to the flux of the reaction that is catalyzed by the enzyme encoded by the particular gene being equal to zero. Analysis of the effect that single or double gene deletions may have on the theoretical yield and maximum ethanol production is important for two reasons: a) it provides insight to the degree to which a particular metabolic reaction or set of two reactions is indeed crucial for cellular growth and/or ethanol production, and b) it enhances the understanding of the metabolic network's connectivity and flexibility to activate alternative routes for the accomplishment of specific biological objectives.

Some of the examined gene deletion LP problems had no feasible solution. In the biological context, these were treated equivalently to the cases in which the optimal value of the objective function was determined equal to zero. For obvious reasons, the deletions of the genes encoding the enzymes that catalyze the catabolic reactions of the two substrates glucose and xylose, r1 and r12-13, respectively, were excluded from this analysis.

##### B.6.1. Effect of single gene deletions on ethanol yield

**Figure 5:**
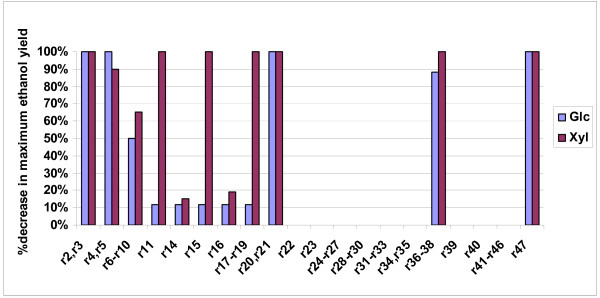
The % decrease in the maximum ethanol yield after the depicted gene's deletion with respect to the original network's for the glucose and xylose substrate cases.

**Figure 6:**
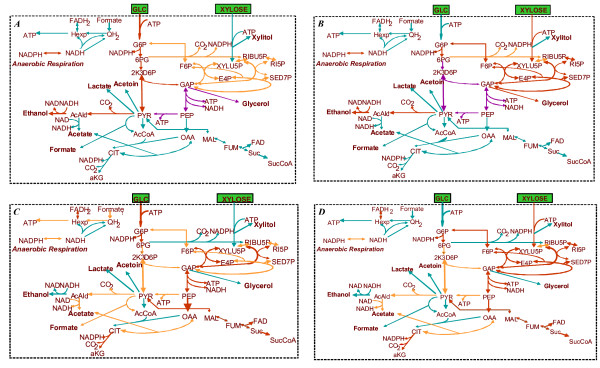
Color-coded metabolic network indicating the impact of each gene's deletion in: A. Maximum ethanol yield when glucose is used as sole substrate; B. Maximum ethanol yield when xylose is used as sole substrate; C. Maximum growth when glucose is used as sole substrate; D. Maximum growth when xylose is used as sole substrate. Green, orange, purple and red color indicate that the deletion of the particular reaction's corresponding gene causes a x% decrease in the optimal objective value, where x = 0, 0 < x < 50, 50 ≤ x < 100 and x = 100, respectively.

In Table 4, the genes are divided into four categories depending on the extent to which the maximum ethanol yield decreases due to each gene's deletion (Figure 5 shows the actual value of the decrease). The four gene categories are subsequently color-coded and depicted in the context of the metabolic network (see Figures 6A–B for glucose and xylose as substrate, respectively), providing thus insight to the importance of each gene's reaction for the network's ethanol producing capability. Studying the solutions of the gene deletion LP problems, the following observations could be made:

**Table 4:** Number of single deletions leading to x% decrease in the maximum ethanol or growth yield

		x = 0	0 < x < 50	50 ≤ x < 100	x = 100
Ethanol Yield	Glucose	22	7	8	7
	Xylose	22	2	7	13

Growth Yield	Glucose	14	14	0	16
	Xylose	17	8	0	19

Both glucose and xylose substrate cases are depicted.

1. Deletion of genes whose reactions are part of the ED, EMP and PP pathways lead to the largest changes in ethanol yield for any of the two utilized substrates. This should be expected, since it is through these pathways that the two substrates are catabolized to PYR and the rest of the products (see section B3). Specifically:

• regarding the ED pathway, when glucose is used as substrate either alone or in combination with xylose, deletion of any gene of the pathway (namely zwf-r2, pgl-r3, edd-r4 or eda-r5) prohibits ethanol production. When xylose is used as substrate, this is true for the deletion of zwf-r2 or pgl-r3. In the case of edd-r4 or eda-r5 deletion, ethanol is produced, but the maximum ethanol yield drops to 10.3% of the maximum for the original network (see section B3) with the simultaneous production of 0.778 mole xylitol per mole of substrate.

• regarding the E.M.P. pathway, deletion of any of its genes (namely gap-r6, pgk-r7, pgm-r8, eno-r9 or pyk-r10) leads to 50% or 65.4% decrease in the maximum capability for the original network to produce ethanol, when the substrate is glucose or xylose, respectively. In all cases, the cell produces 1 mole of glycerol per mole of substrate, while the ATP and NADPH/NADH dual prices are negative, indicating that the ethanol producing capability of the network is constrained by energy requirements. The cell is respiring through r47 (trans), r41 (ndh1), r44 (uqr) and r46 (atpase) reactions.

• regarding the PPP, when glucose is the substrate, deletion of any of the PPP genes (namely pria-r15, rpe-r16, tklb-r17 and r19, tal-r18) and/or gnd-r14 or pgi-r11, the latter two reactions connecting the PPP to the E.D. and EMP pathway, respectively, leads to 11.8% decrease in the maximum capability of the network to produce ethanol. In all cases, the NADPH/NADH dual prices are negative (-0.5), indicating that the network could produce more ethanol should more NADPH or NADH be available. This is true, because the deletion of any of the above mentioned genes results into zero flux for r14, and consequently into production of 1 mole NADPH less than in the original network, while the network produces acetate through r24 (aldh) to satisfy the NADH/NADPH balance constraint. In the case that xylose is used as substrate, deletion of any of the pria-r15, tktlb-r17 and r19, tal-r18 or pgi-r11 prohibits ethanol production. Deletion of rpe-r16 or gnd-r14 leads to 19.2% or 15.4%, respectively, decrease in the network's ethanol producing capability. In the case of the rpe-r16 deletion, the NADH/NADPH dual price is positive (0.25), indicating that if the network could consume more NADPH or NADH through additional metabolic routes, its ethanol producing capability would be higher. This holds true, because XYLU5P cannot be directly converted to RIBU5P, thereby the fluxes through r2 and r14, and consequently the NADPH production rate through these reactions, increase. The cell is thus forced to produce 0.625 mole glycerol/mole xylose to satisfy the zero ATP balance constraint. To respire, the network uses r44 (uqr), r42 (ndh2) and r47 (trans).

2. In the optimal ethanol-producing flux distribution of the original network (see section B.3), glycerol is the only other product apart from ethanol. This justifies the observed strong impact that deletion of any gene in the glycerol biosynthesis pathway (r36-tpi, r37-gpsA, r38) has on the network's ethanol producing capability. Specifically, in the case of glucose substrate, the network's ethanol-producing capability decreased to only 11.8% of the original network's, while it was fully prohibited in the other two substrate cases.

3. The deletion of the trans gene (corresponding to reaction-47) is observed to prohibit ethanol production, in the case that no biosynthetic requirements are taken into consideration, because r-47 is then the only available NADPH sink.

##### B.6.2. Effect of single deletions on cell growth

**Figure 7:**
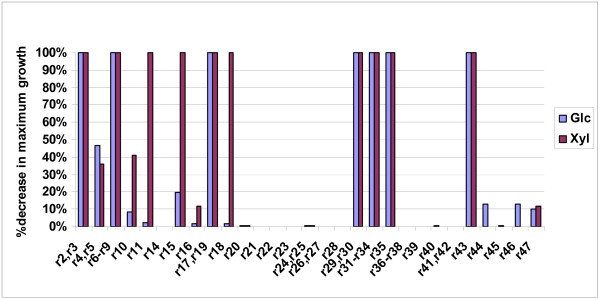
The % decrease in the maximum biomass yield after the depicted gene's deletion with respect to the original network's for the glucose and xylose substrate cases.

In Table 4, the genes are divided into four categories depending on the extent to which maximum growth is decreased due to each gene's deletion (Figure 7 shows the actual value of the decrease after each gene's deletion). In Figures 6C–D, the four color-coded gene categories are depicted in the context of the metabolic network (for glucose and xylose as substrate, respectively), providing thus insight to the importance of each gene's reaction for cell growth. The main observations are summarized below:

1. Deletion of any gene whose reaction's products are biosynthetic precursors is lethal for the cell, if no alternative routes for these compounds' production exist. Indeed, for all examined substrate cases, deletion of any of zwf-r2, pgl-r3, gap-r6, pgk-r7, pgm-r8, eno-r9, aconA-r29, citC-r30, sucD-r33 or ppc-r35 is lethal. In addition, deletion of any gene whose reaction is part of the "right" leg of the TCA cycle, namely fumA-r31, sdhC-r32, yqkJ-r33 or FADH_2 _dehydrogenase (fadh)-r43 (the latter being directly associated with r32), are considered to be lethal, even though they are not used in the optimal solution (see section B.4). These genes are necessary for the production of succinate and subsequently of SucCoA, the latter being used for lysine production (r70). The fact that the production of lysine is accompanied by re-generation of succinate explains why r31, r32, r34 and r43 do not carry flux in the optimal solution, the latter corresponding to the physiology of the cell at metabolic steady-state.

2. Deletion of the PPP tklb-(r17 and r19) gene is lethal for the cell in all examined substrate cases. Deletion of the PPP pria-r15 or PPP tal-r18 or pgi-r11, whose reaction connects the PP to the EMP pathway, are lethal only in the case that xylose is the sole substrate. On the contrary, in the case that glucose is the sole substrate, the deletion of these genes causes only 19.4%, 1.5% and 2.5%, respectively, decrease in the maximum growth.

3. Deletion of EDP edd-r4 and/or eda-r5 leads to 46.8% or 36.1% decrease in the maximum biomass yield, in the case of glucose or xylose, respectively, uptake. Sensitivity analysis indicates that this decrease is due only to the stoichiometric connectivity of the network.

4. The deletion of uqr-r44 and/or atpase-r46 genes in respiration leads to 12.6% decrease in the maximum biomass yield, when glucose is the substrate; it has no impact in the case of xylose substrate, because these two reactions are not used then for cell respiration. Specifically, after these gene deletions in the case of glucose substrate, the cell could respire through multiple equivalent alternative routes, some of which are indicatively discussed here, because they lead to byproduct formation: (i) use of r47(trans) with the simultaneous production of 0.24 mole ethanol; of note, this is the only single gene deletion case, which was observed to lead to ethanol production, (ii) use of r47(trans) with the simultaneous production of 0.24 mole lactate, or (iii) use of r47(trans), r41(ndh1) and r43 (fdhase) with the simultaneous production of 0.2 mole succinate per mole of glucose. Clearly, which of the LP solutions is indeed active *in vivo *can only be determined through comparison with *in vivo Z. mobilis *cell culture data.

##### B.6.3 Effect of double deletions on ethanol yield

In Table 5, the double deletions are divided into four categories depending on the extent to which the maximum ethanol yield is decreased with respect to the original network, for both glucose and xylose substrate cases. Based on the color-code of the single deletions as indicated in Table 4 and Figures 6A–B, Table 5 also shows the single-deletion category of the genes that comprise the double deletions. In the following paragraphs, the double deletions that are mainly discussed lead to greater change in the objective function than the single deletions of the involved genes individually:

**Table 5:** Number of double deletions leading to x% decrease in the maximum ethanol or growth yield

			**x = 0**	**0 < x < 50**		**50 ≤ x < 100**			**x = 100**		
			
		*Single Deletions*	*x = 0*	*x = 0*	*0 < x < 50*	*x = 0*	*0 < x < 50*	*50 ≤ x < 100*	*x = 0*	*0 < x < 50*	*50 ≤ x < 100*
**Ethanol Yield**	**Glc**	*x = 0*	231	0	154	0	0	173	0	0	3
		*0 < x < 50*	N/A		21		0	35		0	21
		*50 ≤ x < 100*	N/A	N/A	N/A			28			0
	**Xyl**	*x = 0*	231	0	44	0	0	142	0	0	12
		*0 < x < 50*	N/A		0		0	5		1	9
		*50 ≤ x < 100*	N/A	N/A	N/A			11			10
**Growth Yield**	**Glc**	*x = 0*	91	0	186	0	1		0	9	
		*0 < x < 50*	N/A		68		1			22	
	**Xyl**	*x = 0*	133	2	117	0	7		1	12	
		*0 < x < 50*	N/A		20		0			8	

Both glucose and xylose substrate cases are depicted. The category of the single deletion of the genes involved in the double deletions with respect to their impact on the objective value (see Table 4) is also depicted.

1. As indicated in B.6.1, in the case that glucose is used as the sole substrate, deletion of any gene in the glycerol biosynthesis pathway (tpi-r36, gpsA-r37 and r38) decreases the network's ethanol-producing capability to only 11.8% of the original network's. Ethanol production is fully prohibited, if these single deletions are combined with either (a) the deletion of the xylitol biosynthesis gene (xyld-r39), which does not cause any change in the network's ethanol producing capability if applied alone, or (b) the deletion of any of the PPP genes (namely pria-r15, rpe-r16, tklb-r17 and r19 and tal-r18) and/or gnd-r14 or pgi-r11, the latter two reactions connecting the PP to the E.D. and EMP pathway, respectively; individual deletion of these genes leads to 11.8% decrease in the maximum ethanol yield (see B.6.1).

2. In the case of xylose catabolism, the simultaneous deletion of gnd-r14, which leads to moderate decrease in the maximum ethanol yield if applied alone (Figure 6), with either (a) any of the E.M.P genes (namely gap-r6, pgk-r7, pgm-r8, eno-r9, pyk-r10), or (b) any of the E.D. genes (namely edd-r4 and eda-r5), all of the above causing significant decrease in the maximum ethanol yield if applied individually (Figures 5, 6B), or (c) the PPP rpe-r16, which is of moderate effect as single deletion (Figures 5, 6B), prohibits ethanol production. The same holds true for the combination of a E.D. gene deletion with either (a) a E.M.P. gene deletion, or (b) the PPP rpe-r16 deletion, or (c) the xyld-r39 deletion.

3. E.M.P. gene deletion could prohibit ethanol production in the case of xylose substrate, if also combined with the respiration's uqr-r44 and/or atpase-r46 gene deletion, the latter two being of no effect to ethanol yield if applied individually (see Figure 5). In the case of glucose catabolism, these double deletions decrease the maximum ethanol yield to 12% of the original network's, while leading to the simultaneous production of 1 mole xylitol per mole glucose to satisfy the equal to zero ATP balance constraint.

##### B.6.4. Effect of double deletions on cell growth

In Table 5, the double deletions are divided into four categories depending on the extent to which the maximum growth is decreased with respect to the original network, for both glucose and xylose substrate cases. Based on the color-code of the single deletions as indicated in Table 4 and Figures 6C–D, Table 5 also shows the single-deletion category of the two genes that comprise the double deletions. In the following paragraphs, only the double deletions that lead to greater change in the objective function than the single deletions of the two involved genes individually are further discussed:

1. Some combinations of gene deletions are lethal as they destroy the alternative routes that the network possesses to produce a biosynthetic precursor; such cases are:(a) the combined deletions of glta-r28 with any of aldh-r24 or cite-r25 or dcp-r20 for both substrate cases, as the biosynthetic precursor αKG cannot be produced; (b) the simultaneous deletion of pfl-r40 with (pdhB, pdhC, lpd)-r22 for both substrate cases, as the cell loses the capability to produce the biosynthetic precursor AcCoA, and (c) the combined deletion of tal-r18 and pria-r15, in the case of glucose consumption (in the case of xylose catabolism, the deletion of any of the two genes is by itself lethal), as there is no other route for the production of RI5P.

2. In the case of glucose catabolism, the simultaneous deletion of any of the E.D. genes (namely edd-r4 and eda-r5) with any of the pyk-r10, pgi-r11, gnd-r14, pria-r15, rpe-r16 or tal-r18 is lethal for the cell. In the case of xylose catabolism, this is true if any of the E.D. pathway genes is deleted simultaneously with pyk-r10, gnd-r14 or rpe-r16. Obviously, when the network loses the ability to catabolize the substrate through the ED pathway, EMP and PP pathways are indispensable for it to grow. Moreover, the simultaneous deletion of gnd-r14, which connects the E.D with the PP pathway, with either pyk-r10 or rpe-r16 in the case of xylose consumption, or with either tal-r18 or pgi-r11 in the case of glucose consumption, are also lethal for the cell. In the case of glucose catabolism, lethal is also the combined deletion of pgi-r11 with either pria-r15 or tal-r18 or rpe-r16 of the PPP.

3. In the case of glucose catabolism, lethal for the cell is the combined deletion of PPP pria-r15 with xyld-r39 of xylitol biosynthesis. In the case of xylose catabolism, lethal due to the network's specific interconnectivity is the double deletion of pyk-r10 with any of tpi-r36, gpsa-r37 or r38 of the glycerol biosynthesis pathway.

4. Lethal for the cell is also the simultaneous deletion of any of the E.D. genes with any of the respiration atpase-r46, uqr-r44 or trans-r47 genes, in the case of glucose substrate. For the xylose catabolism case, only the combined deletion of any of the E.D. pathway genes with the trans-r47 genes is lethal, whereas with any of the uqr-r44 or atpase-r46 genes it leads to 86% decrease in the maximum cell growth. For all these double deletions in both substrate cases, the very high decrease in the objective value originates from the fact that the network loses its capability to convert the produced NADPH/NADH to ATP, in combination with the already considerable decrease that the single deletion of any of the E.D. genes causes to maximum growth (see section B.6.2). This justification is supported by the positive NADH/NADPH dual prices in the two non-lethal deletion cases; in the case of the lethal deletions, the corresponding L.P.'s have no feasible solution.

5. The combined deletion of gnd-r14 and trans-r47, the former of no and the latter of moderate effect as single deletions (see Figures 6C–D, 7), leads to 55% and 64% decrease in the maximum biomass yield, in the case of glucose and xylose substrate, respectively. In this case, the network loses two main NADPH-producing reactions (it can only produce NADPH through r2), which explains the negative NADPH dual price (-58) for both substrate cases.

6. In the case of xylose catabolism, the combined deletion of pyk-r10 (of moderate effect as single deletion; see Figures 6D and 7) and ndh1-r41 (of no effect as single deletion) leads to 50% decrease in the maximum biomass yield. In this case, the network loses the capability to produce ATP through the EMP pathway. In addition, it has to carry flux through r42(ndh2) instead of r41(ndh1), producing thus two protons less and increasing consequently the NADH amount that has to be oxidized for the production of 1 mole ATP. The double deletions of pyk-r10 with any of atpase-r46, trans-r47, uqr-r44 in the case of xylose substrate, or with atpase-r46 or uqr-r44 in the case of glucose consumption, are lethal, because they decrease even further the ability of the network to produce ATP.

7. The combined deletion of rpe-r16 and uqr-r44 (of moderate and no effect, respectively, as single deletions) in the case of xylose as the sole substrate results in 58% decrease in the maximum biomass yield. In addition, the cell has to produce 0.53 mole of xylitol per mole substrate, due to the decrease in the flexibility of the network to consume NADH/NADPH, as indicated from the sensitivity analysis.

8. When xylose is the sole substrate, the simultaneous deletion of trans-r47 and PPP rpe-r16, the latter being of moderate effect on the optimal objective value as single deletions, is lethal as the cell can not produce the required amount of NADPH for the synthesis of biomass precursors. In fact, carrying out the same LP allowing for the NADPH consumption rate to be smaller than its production rate, the maximum biomass yield was 88% of the original network's with NADPH net excretion rate being equal to 0.003 mole per mole of xylose.

9. For the glucose substrate case, the maximum growth rate decreases by 51% after the simultaneous deletion of trans-r47 and rpe-r16. In this case the ATP/NADH dual prices are equal to zero, whereas this of NADPH is negative and equal to -62.12. In this case, the cell has to oxidize the NADH surplus that cannot convert to NADPH through r47 by producing ethanol and/or other by-products in various feasible ratios that are observed as alternative solutions of the L.P. According to these, the cell could potentially produce up to 1 mole ethanol (in this case no other by-product is synthesized). This is a significant result in the context of the analysis for the identification of the most suitable targets for genetic modification towards optimization of ethanol production. It needs further validation through comparison with *in vivo *physiological data. Ethanol production, although small, is also part of the optimal solution(s), in the case of the simultaneous deletion of rpe-r16 with uqr-r44 or atpase-r46, when glucose is the sole substrate; as one of the multiple optimal solutions, the cell could produce 0.09 or 0.16 mole ethanol per mole glucose, respectively, while the maximum biomass yield of the network is decreased only by 12.7% of the original's. These results are among the significant indications of the strong relationship between ethanol production and anaerobic respiration.

## Conclusion

In this paper, the metabolic network of the engineered *Z. mobilis *was reconstructed according to the available published information to a level that it could be modelled based on the existing metabolic engineering methodologies. In sequence, the metabolic boundaries of the microorganism with respect to various biological objectives, including maximization of energy, ethanol and biomass production rate, were determined based only on its metabolic network's stoichiometric connectivity, using linear programming (LP) analysis. Moreover, the impact that the deletion of any gene or combination of two genes could have upon the ethanol producing and growth capabilities of *Z*. *mobilis *were further explored. This study allowed for the identification of the extent to which a given reaction is essential for cellular growth or ethanol production. It also elucidated the connectivity between the various pathways in the network. The major observations include: (a) the ethanol yield is constrained by the fact that the network lacks flexibility to consume the ATP surplus, rendering necessary the biosynthesis of glycerol as competing by-product, and (b) despite growth and ethanol production being competitive for the consumption of the substrate, maximization of growth could be potentially accompanied by ethanol production, sometimes in considerable amount; it was observed that these cases involve the deletion of genes that catalyze reactions of the anaerobic respiration. In general, the results of this study indicated that ethanol and biomass production depend directly on the anaerobic respiration's stoichiometry and activity; thus enhanced understanding and improved means for analyzing anaerobic respiration and redox potential *in vivo *are needed to yield further conclusions for potential genetic targets that may lead to optimized *Z. mobilis *strains. This has indeed been the case in the context of ethanol production from the engineered, to ferment xylose, *S. cerevisiae *[4,15,48]. Taking into consideration that LP is the first level of metabolic network modeling based on stoichiometric connectivity only, the results of the presented study provide significant insight towards the design of experiments, whose data, combined and compared with the simulations, could enhance our understanding of the *in vivo Z. mobilis *ethanol production regulation.

## Materials and methods

### L. P. Model of *Z. mobilis*

A short general description of the LP analysis of metabolic networks is provided in Appendix 1C. Based on this definition, the LP problems addressed in this study were defined as follows.

#### A. Maximization of a metabolite's production rate (biosynthetic requirements are excluded)

The stoichiometric model on which this analysis is based comprises reactions 1–60 in Appendix 1A, involving 60 net fluxes and 59 metabolites. The LP problem to be solved is the following (all reaction and metabolite numbers refer to their listing in Appendices 1A and 1B, respectively):

**Maximize **ZA=rkoutput−rkinput
 MathType@MTEF@5@5@+=feaafiart1ev1aaatCvAUfKttLearuWrP9MDH5MBPbIqV92AaeXatLxBI9gBaebbnrfifHhDYfgasaacH8akY=wiFfYdH8Gipec8Eeeu0xXdbba9frFj0=OqFfea0dXdd9vqai=hGuQ8kuc9pgc9s8qqaq=dirpe0xb9q8qiLsFr0=vr0=vr0dc8meaabaqaciaacaGaaeqabaqabeGadaaakeaacqWGAbGwdaWgaaWcbaGaemyqaeeabeaakiabg2da9iabdkhaYnaaDaaaleaacqWGRbWAaeaacqWGVbWBcqWG1bqDcqWG0baDcqWGWbaCcqWG1bqDcqWG0baDaaGccqGHsislcqWGYbGCdaqhaaWcbaGaem4AaSgabaGaemyAaKMaemOBa4MaemiCaaNaemyDauNaemiDaqhaaaaa@46BE@

subject to:

S¯¯v¯=r¯output−r¯input
MathType@MTEF@5@5@+=feaafiart1ev1aaatCvAUfKttLearuWrP9MDH5MBPbIqV92AaeXatLxBI9gBaebbnrfifHhDYfgasaacH8akY=wiFfYdH8Gipec8Eeeu0xXdbba9frFj0=OqFfea0dXdd9vqai=hGuQ8kuc9pgc9s8qqaq=dirpe0xb9q8qiLsFr0=vr0=vr0dc8meaabaqaciaacaGaaeqabaqabeGadaaakeaadaadbaqaaiabdofatbaadaadaaqaaiabdAha2baacqGH9aqpdaadaaqaaiabdkhaYbaadaahaaWcbeqaaiabd+gaVjabdwha1jabdsha0jabdchaWjabdwha1jabdsha0baakiabgkHiTmaamaaabaGaemOCaihaamaaCaaaleqabaGaemyAaKMaemOBa4MaemiCaaNaemyDauNaemiDaqhaaaaa@4467@     (1a)

vj={≥0j=1−4,10,12,14,20−24,26−28,35,39−42,44−46,48−49,51−60∈Rotherwise     (1b)
 MathType@MTEF@5@5@+=feaafiart1ev1aaatCvAUfKttLearuWrP9MDH5MBPbIqV92AaeXatLxBI9gBaebbnrfifHhDYfgasaacH8akY=wiFfYdH8Gipec8Eeeu0xXdbba9frFj0=OqFfea0dXdd9vqai=hGuQ8kuc9pgc9s8qqaq=dirpe0xb9q8qiLsFr0=vr0=vr0dc8meaabaqaciaacaGaaeqabaqabeGadaaakeaacqWG2bGDdaWgaaWcbaGaemOAaOgabeaakiabg2da9maaceqabaqbaeaabiGaaaqaaiadaciaaWX=gwMiZkadaciaaWX=icdaWaqaauaabiqaceaaaeaacqWGQbGAcqGH9aqpcqaIXaqmcqGHsislcqaI0aancqGGSaalcqaIXaqmcqaIWaamcqGGSaalcqaIXaqmcqaIYaGmcqGGSaalcqaIXaqmcqaI0aancqGGSaalcqaIYaGmcqaIWaamcqGHsislcqaIYaGmcqaI0aancqGGSaalcqaIYaGmcqaI2aGncqGHsislcqaIYaGmcqaI4aaocqGGSaalcqaIZaWmcqaI1aqncqGGSaalaeGabaWqdiabiodaZiabiMda5iabgkHiTiabisda0iabikdaYiabcYcaSiabisda0iabisda0iabgkHiTiabisda0iabiAda2iabcYcaSiabisda0iabiIda4iabgkHiTiabisda0iabiMda5iabcYcaSiabiwda1iabigdaXiabgkHiTiabiAda2iabicdaWaaaaeaacqGHiiIZcqWGsbGuaeaacqqGVbWBcqqG0baDcqqGObaAcqqGLbqzcqqGYbGCcqqG3bWDcqqGPbqAcqqGZbWCcqqGLbqzaaaacaGL7baacaWLjaGaaCzcamaabmaabaGaeGymaeJaeeOyaigacaGLOaGaayzkaaaaaa@7F8D@

r47output−r47input=−a,a∈R+
 MathType@MTEF@5@5@+=feaafiart1ev1aaatCvAUfKttLearuWrP9MDH5MBPbIqV92AaeXatLxBI9gBaebbnrfifHhDYfgasaacH8akY=wiFfYdH8Gipec8Eeeu0xXdbba9frFj0=OqFfea0dXdd9vqai=hGuQ8kuc9pgc9s8qqaq=dirpe0xb9q8qiLsFr0=vr0=vr0dc8meaabaqaciaacaGaaeqabaqabeGadaaakeaafaqabeqacaaabaGaemOCai3aa0baaSqaaiabisda0iabiEda3aqaaiabd+gaVjabdwha1jabdsha0jabdchaWjabdwha1jabdsha0baacqGHsislkiabdkhaYnaaDaaaleaacqaI0aancqaI3aWnaeaacqWGPbqAcqWGUbGBcqWGWbaCcqWG1bqDcqWG0baDaaGccqGH9aqpcqGHsislcqqGHbqycqqGSaalaeaacqqGHbqycqGHiiIZcqqGsbGudaahaaWcbeqaaiabgUcaRaaaaaaaaa@4D99@     (1c)

r48output−r48input=−b,b∈R+
 MathType@MTEF@5@5@+=feaafiart1ev1aaatCvAUfKttLearuWrP9MDH5MBPbIqV92AaeXatLxBI9gBaebbnrfifHhDYfgasaacH8akY=wiFfYdH8Gipec8Eeeu0xXdbba9frFj0=OqFfea0dXdd9vqai=hGuQ8kuc9pgc9s8qqaq=dirpe0xb9q8qiLsFr0=vr0=vr0dc8meaabaqaciaacaGaaeqabaqabeGadaaakeaafaqabeqacaaabaGaemOCai3aa0baaSqaaiabisda0iabiIda4aqaaiabd+gaVjabdwha1jabdsha0jabdchaWjabdwha1jabdsha0baacqGHsislkiabdkhaYnaaDaaaleaacqaI0aancqaI4aaoaeaacqWGPbqAcqWGUbGBcqWGWbaCcqWG1bqDcqWG0baDaaGccqGH9aqpcqGHsislcqWGIbGycqqGSaalaeaaieGacqWFIbGycqGHiiIZcqqGsbGudaahaaWcbeqaaiabgUcaRaaaaaaaaa@4DAC@     (1d)

r49output−r49input=0 or∈R     (1e)
 MathType@MTEF@5@5@+=feaafiart1ev1aaatCvAUfKttLearuWrP9MDH5MBPbIqV92AaeXatLxBI9gBaebbnrfifHhDYfgasaacH8akY=wiFfYdH8Gipec8Eeeu0xXdbba9frFj0=OqFfea0dXdd9vqai=hGuQ8kuc9pgc9s8qqaq=dirpe0xb9q8qiLsFr0=vr0=vr0dc8meaabaqaciaacaGaaeqabaqabeGadaaakeaacqWGYbGCdaqhaaWcbaGaeGinaqJaeGyoaKdabaGaem4Ba8MaemyDauNaemiDaqNaemiCaaNaemyDauNaemiDaqhaaOGaeyOeI0IaemOCai3aa0baaSqaaiabisda0iabiMda5aqaaiabdMgaPjabd6gaUjabdchaWjabdwha1jabdsha0baakiabg2da9iabicdaWiabbccaGiabd+gaVjabdkhaYjabgIGiolabdkfasjaaxMaacaWLjaWaaeWaaeaacqaIXaqmcqqGLbqzaiaawIcacaGLPaaaaaa@51C0@

r50output−r50input∈R
 MathType@MTEF@5@5@+=feaafiart1ev1aaatCvAUfKttLearuWrP9MDH5MBPbIqV92AaeXatLxBI9gBaebbnrfifHhDYfgasaacH8akY=wiFfYdH8Gipec8Eeeu0xXdbba9frFj0=OqFfea0dXdd9vqai=hGuQ8kuc9pgc9s8qqaq=dirpe0xb9q8qiLsFr0=vr0=vr0dc8meaabaqaciaacaGaaeqabaqabeGadaaakeaacqWGYbGCdaqhaaWcbaGaeGynauJaeGimaadabaGaem4Ba8MaemyDauNaemiDaqNaemiCaaNaemyDauNaemiDaqhaaOGaeyOeI0IaemOCai3aa0baaSqaaiabiwda1iabicdaWaqaaiabdMgaPjabd6gaUjabdchaWjabdwha1jabdsha0baakiabgIGiolabdkfasbaa@4703@     (1f)

rqoutput−rqinput∈R+for q=51−59
 MathType@MTEF@5@5@+=feaafiart1ev1aaatCvAUfKttLearuWrP9MDH5MBPbIqV92AaeXatLxBI9gBaebbnrfifHhDYfgasaacH8akY=wiFfYdH8Gipec8Eeeu0xXdbba9frFj0=OqFfea0dXdd9vqai=hGuQ8kuc9pgc9s8qqaq=dirpe0xb9q8qiLsFr0=vr0=vr0dc8meaabaqaciaacaGaaeqabaqabeGadaaakeaafaqabeqacaaabaGaemOCai3aa0baaSqaaiabdghaXbqaaiabd+gaVjabdwha1jabdsha0jabdchaWjabdwha1jabdsha0baakiabgkHiTiabdkhaYnaaDaaaleaacqWGXbqCaeaacqWGPbqAcqWGUbGBcqWGWbaCcqWG1bqDcqWG0baDaaGccqGHiiIZcqWGsbGudaahaaWcbeqaaiabgUcaRaaaaOqaaiabbAgaMjabb+gaVjabbkhaYjabbccaGiabbghaXHGaaiab=1da9iabbwda1iabbgdaXiab=jHiTiabbwda1iabbMda5aaaaaa@5339@     (1g)

rqoutput−rqinput=0for q=1−46     (1h)
 MathType@MTEF@5@5@+=feaafiart1ev1aaatCvAUfKttLearuWrP9MDH5MBPbIqV92AaeXatLxBI9gBaebbnrfifHhDYfgasaacH8akY=wiFfYdH8Gipec8Eeeu0xXdbba9frFj0=OqFfea0dXdd9vqai=hGuQ8kuc9pgc9s8qqaq=dirpe0xb9q8qiLsFr0=vr0=vr0dc8meaabaqaciaacaGaaeqabaqabeGadaaakeaafaqabeqacaaabaGaemOCai3aa0baaSqaaiabdghaXbqaaiabd+gaVjabdwha1jabdsha0jabdchaWjabdwha1jabdsha0baakiabgkHiTiabdkhaYnaaDaaaleaacqWGXbqCaeaacqWGPbqAcqWGUbGBcqWGWbaCcqWG1bqDcqWG0baDaaGccqGH9aqpcqaIWaamaeaacqqGMbGzcqqGVbWBcqqGYbGCcqqGGaaicqqGXbqCiiaacqWF9aqpcqqGXaqmcqWFsislcqqG0aancqqG2aGnaaGaaCzcaiaaxMaadaqadaqaaiabigdaXiabbIgaObGaayjkaiaawMcaaaaa@557E@

(*v*_*j *_= 0 in the case that the gene which encodes the enzyme that catalyzes reaction j is deleted)

where:

*Z*_*A *_the objective function

k the Appendix 1B number of metabolite whose production rate is to be optimized

rioutput−riinput
 MathType@MTEF@5@5@+=feaafiart1ev1aaatCvAUfKttLearuWrP9MDH5MBPbIqV92AaeXatLxBI9gBaebbnrfifHhDYfgasaacH8akY=wiFfYdH8Gipec8Eeeu0xXdbba9frFj0=OqFfea0dXdd9vqai=hGuQ8kuc9pgc9s8qqaq=dirpe0xb9q8qiLsFr0=vr0=vr0dc8meaabaqaciaacaGaaeqabaqabeGadaaakeaacqWGYbGCdaqhaaWcbaGaemyAaKgabaGaem4Ba8MaemyDauNaemiDaqNaemiCaaNaemyDauNaemiDaqhaaOGaeyOeI0IaemOCai3aa0baaSqaaiabdMgaPbqaaiabdMgaPjabd6gaUjabdchaWjabdwha1jabdsha0baaaaa@4332@ the net excretion rate of the i-th metabolite

S¯¯
MathType@MTEF@5@5@+=feaafiart1ev1aaatCvAUfKttLearuWrP9MDH5MBPbIqV92AaeXatLxBI9gBaebbnrfifHhDYfgasaacH8akY=wiFfYdH8Gipec8Eeeu0xXdbba9frFj0=OqFfea0dXdd9vqai=hGuQ8kuc9pgc9s8qqaq=dirpe0xb9q8qiLsFr0=vr0=vr0dc8meaabaqaciaacaGaaeqabaqabeGadaaakeaadaadbaqaaiabdofatbaaaaa@2DEC@ the 59(|# of metabolites) × 60(# of reactions) stoichiometric matrix of the metabolic network (in this case, each reaction is represented by the stoichiometry of the direction in which it is depicted in Appendix 1A, i.e. this has been selected as the default direction of its net flux)

v¯
 MathType@MTEF@5@5@+=feaafiart1ev1aqatCvAUfKttLearuWrP9MDH5MBPbIqV92AaeXatLxBI9gBaebbnrfifHhDYfgasaacH8akY=wiFfYdH8Gipec8Eeeu0xXdbba9frFj0=OqFfea0dXdd9vqai=hGuQ8kuc9pgc9s8qqaq=dirpe0xb9q8qiLsFr0=vr0=vr0dc8meaabaqaciaacaGaaeqabaqabeGadaaakeaadaadaaqaaiabdAha2baaaaa@2E32@ the 60 × 1 metabolic net flux vector (see comment above about the default direction of a reaction's net flux – if the LP optimal solution corresponds to a negative value for the j-th net flux, this indicates that its direction is opposite than the default)

(1α) Metabolite balance constraints

(1b) The non-negative constraint on the net fluxes of the irreversible reactions

(1c), (1d) The three examined substrate cases were: a = 1 and b = 0, a = 0 and b = 1, a = 0.5 and b = 0.5. Due to the linearity of problem, the solution of the latter case is an interpolation of the first two. Similarly, for any values of a and b, the solution of the problem will be the weighted interpolation of the solutions of the first two cases (i.e. glucose or xylose, as sole substrates).

(1e) Regarding the net excretion rate of ATP, two cases were examined: (a) rATPoutput−rATPinput=0
 MathType@MTEF@5@5@+=feaafiart1ev1aaatCvAUfKttLearuWrP9MDH5MBPbIqV92AaeXatLxBI9gBaebbnrfifHhDYfgasaacH8akY=wiFfYdH8Gipec8Eeeu0xXdbba9frFj0=OqFfea0dXdd9vqai=hGuQ8kuc9pgc9s8qqaq=dirpe0xb9q8qiLsFr0=vr0=vr0dc8meaabaqaciaacaGaaeqabaqabeGadaaakeaacqWGYbGCdaqhaaWcbaGaemyqaeKaemivaqLaemiuaafabaGaem4Ba8MaemyDauNaemiDaqNaemiCaaNaemyDauNaemiDaqhaaOGaeyOeI0IaemOCai3aa0baaSqaaiabdgeabjabdsfaujabdcfaqbqaaiabdMgaPjabd6gaUjabdchaWjabdwha1jabdsha0baakiabg2da9iabicdaWaaa@4944@, and (b) no constraint on the ATP net excretion rate was imposed.

(1f) No constraint was imposed on the *CO*_2 _net excretion rate.

(1g) The net excretion rates of all potential products are considered as nonnegative

(1h) The net excretion rates of all intracellular metabolites are considered equal to 0

#### B. Maximization of cellular growth rate

The stoichiometric model on which this analysis is based comprises reactions 1–79 in Appendix 1A, involving 79 net fluxes and 77 metabolites. The LP problem to be solved is the following:

**Maximize ***Z*_*b *_= *v*_79_

subject to:

S¯¯v¯=r¯output−r¯input
MathType@MTEF@5@5@+=feaafiart1ev1aaatCvAUfKttLearuWrP9MDH5MBPbIqV92AaeXatLxBI9gBaebbnrfifHhDYfgasaacH8akY=wiFfYdH8Gipec8Eeeu0xXdbba9frFj0=OqFfea0dXdd9vqai=hGuQ8kuc9pgc9s8qqaq=dirpe0xb9q8qiLsFr0=vr0=vr0dc8meaabaqaciaacaGaaeqabaqabeGadaaakeaadaadbaqaaiabdofatbaadaadaaqaaiabdAha2baacqGH9aqpdaadaaqaaiabdkhaYbaadaahaaWcbeqaaiabd+gaVjabdwha1jabdsha0jabdchaWjabdwha1jabdsha0baakiabgkHiTmaamaaabaGaemOCaihaamaaCaaaleqabaGaemyAaKMaemOBa4MaemiCaaNaemyDauNaemiDaqhaaaaa@4467@     (2a)

vj={≥0j=1−4,10,12,14,20−24,26−28,35,39−42,44−46,48−49,51−79∈Rotherwise
 MathType@MTEF@5@5@+=feaafiart1ev1aaatCvAUfKttLearuWrP9MDH5MBPbIqV92AaeXatLxBI9gBaebbnrfifHhDYfgasaacH8akY=wiFfYdH8Gipec8Eeeu0xXdbba9frFj0=OqFfea0dXdd9vqai=hGuQ8kuc9pgc9s8qqaq=dirpe0xb9q8qiLsFr0=vr0=vr0dc8meaabaqaciaacaGaaeqabaqabeGadaaakeaacqWG2bGDdaWgaaWcbaGaemOAaOgabeaakiabg2da9maaceqabaqbaeaabiGaaaqaaiadaciaaqW=gwMiZkadaciaaqW=icdaWaqaauaabiqaceaaaeaacqWGQbGAcqGH9aqpcqaIXaqmcqGHsislcqaI0aancqGGSaalcqaIXaqmcqaIWaamcqGGSaalcqaIXaqmcqaIYaGmcqGGSaalcqaIXaqmcqaI0aancqGGSaalcqaIYaGmcqaIWaamcqGHsislcqaIYaGmcqaI0aancqGGSaalcqaIYaGmcqaI2aGncqGHsislcqaIYaGmcqaI4aaocqGGSaalcqaIZaWmcqaI1aqncqGGSaalaeaacqaIZaWmcqaI5aqocqGHsislcqaI0aancqaIYaGmcqGGSaalcqaI0aancqaI0aancqGHsislcqaI0aancqaI2aGncqGGSaalcqaI0aancqaI4aaocqGHsislcqaI0aancqaI5aqocqGGSaalcqaI1aqncqaIXaqmcqGHsislcqaI3aWncqaI5aqoaaaabaGaeyicI4SaemOuaifabaGaee4Ba8MaeeiDaqNaeeiAaGMaeeyzauMaeeOCaiNaee4DaCNaeeyAaKMaee4CamNaeeyzaugaaaGaay5Eaaaaaa@7A20@     (2b)

r47output−r47input=−a,a∈R+
 MathType@MTEF@5@5@+=feaafiart1ev1aaatCvAUfKttLearuWrP9MDH5MBPbIqV92AaeXatLxBI9gBaebbnrfifHhDYfgasaacH8akY=wiFfYdH8Gipec8Eeeu0xXdbba9frFj0=OqFfea0dXdd9vqai=hGuQ8kuc9pgc9s8qqaq=dirpe0xb9q8qiLsFr0=vr0=vr0dc8meaabaqaciaacaGaaeqabaqabeGadaaakeaafaqabeqacaaabaGaemOCai3aa0baaSqaaiabisda0iabiEda3aqaaiabd+gaVjabdwha1jabdsha0jabdchaWjabdwha1jabdsha0baacqGHsislkiabdkhaYnaaDaaaleaacqaI0aancqaI3aWnaeaacqWGPbqAcqWGUbGBcqWGWbaCcqWG1bqDcqWG0baDaaGccqGH9aqpcqGHsislcqqGHbqycqqGSaalaeaacqqGHbqycqGHiiIZcqqGsbGudaahaaWcbeqaaiabgUcaRaaaaaaaaa@4D99@     (2c)

r48output−r48input=−b,b∈R+
 MathType@MTEF@5@5@+=feaafiart1ev1aaatCvAUfKttLearuWrP9MDH5MBPbIqV92AaeXatLxBI9gBaebbnrfifHhDYfgasaacH8akY=wiFfYdH8Gipec8Eeeu0xXdbba9frFj0=OqFfea0dXdd9vqai=hGuQ8kuc9pgc9s8qqaq=dirpe0xb9q8qiLsFr0=vr0=vr0dc8meaabaqaciaacaGaaeqabaqabeGadaaakeaafaqabeqacaaabaGaemOCai3aa0baaSqaaiabisda0iabiIda4aqaaiabd+gaVjabdwha1jabdsha0jabdchaWjabdwha1jabdsha0baacqGHsislkiabdkhaYnaaDaaaleaacqaI0aancqaI4aaoaeaacqWGPbqAcqWGUbGBcqWGWbaCcqWG1bqDcqWG0baDaaGccqGH9aqpcqGHsislcqWGIbGycqqGSaalaeaaieGacqWFIbGycqGHiiIZcqqGsbGudaahaaWcbeqaaiabgUcaRaaaaaaaaa@4DAC@     (2d)

r49output−r49input∈R+
 MathType@MTEF@5@5@+=feaafiart1ev1aaatCvAUfKttLearuWrP9MDH5MBPbIqV92AaeXatLxBI9gBaebbnrfifHhDYfgasaacH8akY=wiFfYdH8Gipec8Eeeu0xXdbba9frFj0=OqFfea0dXdd9vqai=hGuQ8kuc9pgc9s8qqaq=dirpe0xb9q8qiLsFr0=vr0=vr0dc8meaabaqaciaacaGaaeqabaqabeGadaaakeaacqWGYbGCdaqhaaWcbaGaeGinaqJaeGyoaKdabaGaem4Ba8MaemyDauNaemiDaqNaemiCaaNaemyDauNaemiDaqhaaOGaeyOeI0IaemOCai3aa0baaSqaaiabisda0iabiMda5aqaaiabdMgaPjabd6gaUjabdchaWjabdwha1jabdsha0baakiabgIGiolabbkfasnaaCaaaleqabaGaey4kaScaaaaa@4830@     (2e)

r50output−r50input∈R
 MathType@MTEF@5@5@+=feaafiart1ev1aaatCvAUfKttLearuWrP9MDH5MBPbIqV92AaeXatLxBI9gBaebbnrfifHhDYfgasaacH8akY=wiFfYdH8Gipec8Eeeu0xXdbba9frFj0=OqFfea0dXdd9vqai=hGuQ8kuc9pgc9s8qqaq=dirpe0xb9q8qiLsFr0=vr0=vr0dc8meaabaqaciaacaGaaeqabaqabeGadaaakeaacqWGYbGCdaqhaaWcbaGaeGynauJaeGimaadabaGaem4Ba8MaemyDauNaemiDaqNaemiCaaNaemyDauNaemiDaqhaaOGaeyOeI0IaemOCai3aa0baaSqaaiabiwda1iabicdaWaqaaiabdMgaPjabd6gaUjabdchaWjabdwha1jabdsha0baakiabgIGiolabdkfasbaa@4703@     (2f)

rqoutput−rqinput∈R+for q=51−59
 MathType@MTEF@5@5@+=feaafiart1ev1aaatCvAUfKttLearuWrP9MDH5MBPbIqV92AaeXatLxBI9gBaebbnrfifHhDYfgasaacH8akY=wiFfYdH8Gipec8Eeeu0xXdbba9frFj0=OqFfea0dXdd9vqai=hGuQ8kuc9pgc9s8qqaq=dirpe0xb9q8qiLsFr0=vr0=vr0dc8meaabaqaciaacaGaaeqabaqabeGadaaakeaafaqabeqacaaabaGaemOCai3aa0baaSqaaiabdghaXbqaaiabd+gaVjabdwha1jabdsha0jabdchaWjabdwha1jabdsha0baakiabgkHiTiabdkhaYnaaDaaaleaacqWGXbqCaeaacqWGPbqAcqWGUbGBcqWGWbaCcqWG1bqDcqWG0baDaaGccqGHiiIZcqWGsbGudaahaaWcbeqaaiabgUcaRaaaaOqaaiabbAgaMjabb+gaVjabbkhaYjabbccaGiabbghaXHGaaiab=1da9iabbwda1iabbgdaXiab=jHiTiabbwda1iabbMda5aaaaaa@5339@     (2g)

rqoutput−rqinput=0for q=1−46,60−77
 MathType@MTEF@5@5@+=feaafiart1ev1aaatCvAUfKttLearuWrP9MDH5MBPbIqV92AaeXatLxBI9gBaebbnrfifHhDYfgasaacH8akY=wiFfYdH8Gipec8Eeeu0xXdbba9frFj0=OqFfea0dXdd9vqai=hGuQ8kuc9pgc9s8qqaq=dirpe0xb9q8qiLsFr0=vr0=vr0dc8meaabaqaciaacaGaaeqabaqabeGadaaakeaafaqabeqacaaabaGaemOCai3aa0baaSqaaiabdghaXbqaaiabd+gaVjabdwha1jabdsha0jabdchaWjabdwha1jabdsha0baakiabgkHiTiabdkhaYnaaDaaaleaacqWGXbqCaeaacqWGPbqAcqWGUbGBcqWGWbaCcqWG1bqDcqWG0baDaaGccqGH9aqpcqaIWaamaeaacqqGMbGzcqqGVbWBcqqGYbGCcqqGGaaicqqGXbqCcqGH9aqpcqaIXaqmcqGHsislcqaI0aancqaI2aGncqGGSaalcqaI2aGncqaIWaamcqGHsislcqaI3aWncqaI3aWnaaaaaa@5630@     (2h)

where:

S¯¯
MathType@MTEF@5@5@+=feaafiart1ev1aaatCvAUfKttLearuWrP9MDH5MBPbIqV92AaeXatLxBI9gBaebbnrfifHhDYfgasaacH8akY=wiFfYdH8Gipec8Eeeu0xXdbba9frFj0=OqFfea0dXdd9vqai=hGuQ8kuc9pgc9s8qqaq=dirpe0xb9q8qiLsFr0=vr0=vr0dc8meaabaqaciaacaGaaeqabaqabeGadaaakeaadaadbaqaaiabdofatbaaaaa@2DEC@ the 77(|# of metabolites) × 79(# of reactions) stoichiometric matrix of the metabolic network

All other symbols are defined as in the L.P. described in section A [L.P. (1)].

Constraints (2a)-(2d), (2f)-(2h) are defined as in L.P (1). Constraint (2e) describes the assumption that the ATP produced from the network is at least as much as the ATP consumed.

#### C. Maximization of a metabolite's production rate taking into consideration the biosynthetic requirements

The stoichiometric model is the same as in section B [LP(2)]. The L.P. problem to be solved is the following:

**Maximize **Zc=rkoutput−rkinput
 MathType@MTEF@5@5@+=feaafiart1ev1aaatCvAUfKttLearuWrP9MDH5MBPbIqV92AaeXatLxBI9gBaebbnrfifHhDYfgasaacH8akY=wiFfYdH8Gipec8Eeeu0xXdbba9frFj0=OqFfea0dXdd9vqai=hGuQ8kuc9pgc9s8qqaq=dirpe0xb9q8qiLsFr0=vr0=vr0dc8meaabaqaciaacaGaaeqabaqabeGadaaakeaacqWGAbGwdaWgaaWcbaGaem4yamgabeaakiabg2da9iabdkhaYnaaDaaaleaacqWGRbWAaeaacqWGVbWBcqWG1bqDcqWG0baDcqWGWbaCcqWG1bqDcqWG0baDaaGccqGHsislcqWGYbGCdaqhaaWcbaGaem4AaSgabaGaemyAaKMaemOBa4MaemiCaaNaemyDauNaemiDaqhaaaaa@4702@

subject to:

S¯¯v¯=r¯output−r¯input
MathType@MTEF@5@5@+=feaafiart1ev1aaatCvAUfKttLearuWrP9MDH5MBPbIqV92AaeXatLxBI9gBaebbnrfifHhDYfgasaacH8akY=wiFfYdH8Gipec8Eeeu0xXdbba9frFj0=OqFfea0dXdd9vqai=hGuQ8kuc9pgc9s8qqaq=dirpe0xb9q8qiLsFr0=vr0=vr0dc8meaabaqaciaacaGaaeqabaqabeGadaaakeaadaadbaqaaiabdofatbaadaadaaqaaiabdAha2baacqGH9aqpdaadaaqaaiabdkhaYbaadaahaaWcbeqaaiabd+gaVjabdwha1jabdsha0jabdchaWjabdwha1jabdsha0baakiabgkHiTmaamaaabaGaemOCaihaamaaCaaaleqabaGaemyAaKMaemOBa4MaemiCaaNaemyDauNaemiDaqhaaaaa@4467@     (3a)

vj={≥0j=1−4,10,12,14,20−24,26−28,35,39−42,44−46,48−49,51−79∈Rotherwise
 MathType@MTEF@5@5@+=feaafiart1ev1aaatCvAUfKttLearuWrP9MDH5MBPbIqV92AaeXatLxBI9gBaebbnrfifHhDYfgasaacH8akY=wiFfYdH8Gipec8Eeeu0xXdbba9frFj0=OqFfea0dXdd9vqai=hGuQ8kuc9pgc9s8qqaq=dirpe0xb9q8qiLsFr0=vr0=vr0dc8meaabaqaciaacaGaaeqabaqabeGadaaakeaacqWG2bGDdaWgaaWcbaGaemOAaOgabeaakiabg2da9maaceqabaqbaeaabiGaaaqaaiadaciaaqW=gwMiZkadaciaaqW=icdaWaqaauaabiqaceaaaeaacqWGQbGAcqGH9aqpcqaIXaqmcqGHsislcqaI0aancqGGSaalcqaIXaqmcqaIWaamcqGGSaalcqaIXaqmcqaIYaGmcqGGSaalcqaIXaqmcqaI0aancqGGSaalcqaIYaGmcqaIWaamcqGHsislcqaIYaGmcqaI0aancqGGSaalcqaIYaGmcqaI2aGncqGHsislcqaIYaGmcqaI4aaocqGGSaalcqaIZaWmcqaI1aqncqGGSaalaeaacqaIZaWmcqaI5aqocqGHsislcqaI0aancqaIYaGmcqGGSaalcqaI0aancqaI0aancqGHsislcqaI0aancqaI2aGncqGGSaalcqaI0aancqaI4aaocqGHsislcqaI0aancqaI5aqocqGGSaalcqaI1aqncqaIXaqmcqGHsislcqaI3aWncqaI5aqoaaaabaGaeyicI4SaemOuaifabaGaee4Ba8MaeeiDaqNaeeiAaGMaeeyzauMaeeOCaiNaee4DaCNaeeyAaKMaee4CamNaeeyzaugaaaGaay5Eaaaaaa@7A20@     (3b)

r47output−r47input=−a,a∈R+
 MathType@MTEF@5@5@+=feaafiart1ev1aaatCvAUfKttLearuWrP9MDH5MBPbIqV92AaeXatLxBI9gBaebbnrfifHhDYfgasaacH8akY=wiFfYdH8Gipec8Eeeu0xXdbba9frFj0=OqFfea0dXdd9vqai=hGuQ8kuc9pgc9s8qqaq=dirpe0xb9q8qiLsFr0=vr0=vr0dc8meaabaqaciaacaGaaeqabaqabeGadaaakeaafaqabeqacaaabaGaemOCai3aa0baaSqaaiabisda0iabiEda3aqaaiabd+gaVjabdwha1jabdsha0jabdchaWjabdwha1jabdsha0baacqGHsislkiabdkhaYnaaDaaaleaacqaI0aancqaI3aWnaeaacqWGPbqAcqWGUbGBcqWGWbaCcqWG1bqDcqWG0baDaaGccqGH9aqpcqGHsislcqqGHbqycqqGSaalaeaacqqGHbqycqGHiiIZcqqGsbGudaahaaWcbeqaaiabgUcaRaaaaaaaaa@4D99@     (3c)

r48output−r48input=−b,b∈R+
 MathType@MTEF@5@5@+=feaafiart1ev1aaatCvAUfKttLearuWrP9MDH5MBPbIqV92AaeXatLxBI9gBaebbnrfifHhDYfgasaacH8akY=wiFfYdH8Gipec8Eeeu0xXdbba9frFj0=OqFfea0dXdd9vqai=hGuQ8kuc9pgc9s8qqaq=dirpe0xb9q8qiLsFr0=vr0=vr0dc8meaabaqaciaacaGaaeqabaqabeGadaaakeaafaqabeqacaaabaGaemOCai3aa0baaSqaaiabisda0iabiIda4aqaaiabd+gaVjabdwha1jabdsha0jabdchaWjabdwha1jabdsha0baacqGHsislkiabdkhaYnaaDaaaleaacqaI0aancqaI4aaoaeaacqWGPbqAcqWGUbGBcqWGWbaCcqWG1bqDcqWG0baDaaGccqGH9aqpcqGHsislcqWGIbGycqqGSaalaeaaieGacqWFIbGycqGHiiIZcqqGsbGudaahaaWcbeqaaiabgUcaRaaaaaaaaa@4DAC@     (3d)

r49output−r49input∈R+
 MathType@MTEF@5@5@+=feaafiart1ev1aaatCvAUfKttLearuWrP9MDH5MBPbIqV92AaeXatLxBI9gBaebbnrfifHhDYfgasaacH8akY=wiFfYdH8Gipec8Eeeu0xXdbba9frFj0=OqFfea0dXdd9vqai=hGuQ8kuc9pgc9s8qqaq=dirpe0xb9q8qiLsFr0=vr0=vr0dc8meaabaqaciaacaGaaeqabaqabeGadaaakeaacqWGYbGCdaqhaaWcbaGaeGinaqJaeGyoaKdabaGaem4Ba8MaemyDauNaemiDaqNaemiCaaNaemyDauNaemiDaqhaaOGaeyOeI0IaemOCai3aa0baaSqaaiabisda0iabiMda5aqaaiabdMgaPjabd6gaUjabdchaWjabdwha1jabdsha0baakiabgIGiolabbkfasnaaCaaaleqabaGaey4kaScaaaaa@4830@     (3e)

r50output−r50input∈R
 MathType@MTEF@5@5@+=feaafiart1ev1aaatCvAUfKttLearuWrP9MDH5MBPbIqV92AaeXatLxBI9gBaebbnrfifHhDYfgasaacH8akY=wiFfYdH8Gipec8Eeeu0xXdbba9frFj0=OqFfea0dXdd9vqai=hGuQ8kuc9pgc9s8qqaq=dirpe0xb9q8qiLsFr0=vr0=vr0dc8meaabaqaciaacaGaaeqabaqabeGadaaakeaacqWGYbGCdaqhaaWcbaGaeGynauJaeGimaadabaGaem4Ba8MaemyDauNaemiDaqNaemiCaaNaemyDauNaemiDaqhaaOGaeyOeI0IaemOCai3aa0baaSqaaiabiwda1iabicdaWaqaaiabdMgaPjabd6gaUjabdchaWjabdwha1jabdsha0baakiabgIGiolabdkfasbaa@4703@     (3f)

rqoutput−rqinput∈R+for q=51−59
 MathType@MTEF@5@5@+=feaafiart1ev1aaatCvAUfKttLearuWrP9MDH5MBPbIqV92AaeXatLxBI9gBaebbnrfifHhDYfgasaacH8akY=wiFfYdH8Gipec8Eeeu0xXdbba9frFj0=OqFfea0dXdd9vqai=hGuQ8kuc9pgc9s8qqaq=dirpe0xb9q8qiLsFr0=vr0=vr0dc8meaabaqaciaacaGaaeqabaqabeGadaaakeaafaqabeqacaaabaGaemOCai3aa0baaSqaaiabdghaXbqaaiabd+gaVjabdwha1jabdsha0jabdchaWjabdwha1jabdsha0baakiabgkHiTiabdkhaYnaaDaaaleaacqWGXbqCaeaacqWGPbqAcqWGUbGBcqWGWbaCcqWG1bqDcqWG0baDaaGccqGHiiIZcqWGsbGudaahaaWcbeqaaiabgUcaRaaaaOqaaiabbAgaMjabb+gaVjabbkhaYjabbccaGiabbghaXHGaaiab=1da9iabbwda1iabbgdaXiab=jHiTiabbwda1iabbMda5aaaaaa@5339@     (3g)

rqoutput−rqinput=0for q=1−46,60−77
 MathType@MTEF@5@5@+=feaafiart1ev1aaatCvAUfKttLearuWrP9MDH5MBPbIqV92AaeXatLxBI9gBaebbnrfifHhDYfgasaacH8akY=wiFfYdH8Gipec8Eeeu0xXdbba9frFj0=OqFfea0dXdd9vqai=hGuQ8kuc9pgc9s8qqaq=dirpe0xb9q8qiLsFr0=vr0=vr0dc8meaabaqaciaacaGaaeqabaqabeGadaaakeaafaqabeqacaaabaGaemOCai3aa0baaSqaaiabdghaXbqaaiabd+gaVjabdwha1jabdsha0jabdchaWjabdwha1jabdsha0baakiabgkHiTiabdkhaYnaaDaaaleaacqWGXbqCaeaacqWGPbqAcqWGUbGBcqWGWbaCcqWG1bqDcqWG0baDaaGccqGH9aqpcqaIWaamaeaacqqGMbGzcqqGVbWBcqqGYbGCcqqGGaaicqqGXbqCcqGH9aqpcqaIXaqmcqGHsislcqaI0aancqaI2aGncqGGSaalcqaI2aGncqaIWaamcqGHsislcqaI3aWncqaI3aWnaaaaaa@5630@     (3h)

v_79 _= β(max *Z*_*B*_) 0 < β < 1     (3i)

(*v*_*j *_= 0 in the case that the gene which encodes for the enzyme that catalyzes reaction j is deleted)

All symbols are defined as in section B. Constraints (3a)-(3h) are the same as in LP(2). Constraint (3i) describes the case in which the growth rate is constrained to be equal to a fraction β of the theoretical maximum (i.e. the solution of LP(2)).

All LP problems of this study were solved using the Solver tool of Microsoft Excel (Microsoft Office 2003).

• The dual price of metabolite i represents the change in the optimal value of the cellular objective function due to the by 1 unit increase in the net excretion rate of metabolite i [36,37,49-51];

• When the capability of the network to produce any of the biosynthetic precursors is being determined, the C conversion for each precursor is also estimated. C conversion is defined as follows [36]: Cconversion=(#)C atoms of the precursor(#)C atoms of the substrate×yield
 MathType@MTEF@5@5@+=feaafiart1ev1aaatCvAUfKttLearuWrP9MDH5MBPbIqV92AaeXatLxBI9gBaebbnrfifHhDYfgasaacH8akY=wiFfYdH8Gipec8Eeeu0xXdbba9frFj0=OqFfea0dXdd9vqai=hGuQ8kuc9pgc9s8qqaq=dirpe0xb9q8qiLsFr0=vr0=vr0dc8meaabaqaciaacaGaaeqabaqabeGadaaakeaacqqGdbWqcqqGJbWycqqGVbWBcqqGUbGBcqqG2bGDcqqGLbqzcqqGYbGCcqqGZbWCcqqGPbqAcqqGVbWBcqqGUbGBcqGH9aqpdaWcaaqaaiabbIcaOiabbocaJiabbMcaPiabboeadjabbccaGiabbggaHjabbsha0jabb+gaVjabb2gaTjabbohaZjabbccaGiabb+gaVjabbAgaMjabbccaGiabbsha0jabbIgaOjabbwgaLjabbccaGiabbchaWjabbkhaYjabbwgaLjabbogaJjabbwha1jabbkhaYjabbohaZjabb+gaVjabbkhaYbqaaiabbIcaOiabbocaJiabbMcaPiabboeadjabbccaGiabbggaHjabbsha0jabb+gaVjabb2gaTjabbohaZjabbccaGiabb+gaVjabbAgaMjabbccaGiabbsha0jabbIgaOjabbwgaLjabbccaGiabbohaZjabbwha1jabbkgaIjabbohaZjabbsha0jabbkhaYjabbggaHjabbsha0jabbwgaLbaacqGHxdaTcqqG5bqEcqqGPbqAcqqGLbqzcqqGSbaBcqqGKbazaaa@874B@ and represents the capability to convert the C atoms of the substrate to the C atoms of the desired precursor.

• C conversion in combination with the ATP dual price indicates whether stoichoiometry or energy requirements limit the full conversion of the substrate into any of the precursors [36]. In the case that the ATP dual price is zero and the C conversion is smaller than 100%, it is concluded that a higher precursor yield is constrained only by the stoichiometry of the network. In the case that the ATP dual price is negative then a higher precursor yield is constrained by the energy requirements. In the case that the ATP dual price is positive, then a higher precursor yield is constrained by the fact that the network lacks flexibility to consume the ATP surplus. If C conversion remains smaller than 100% even in the case that the ATP balance constraint is not taken into consideration in the LP problem, then for a nonzero ATP dual price a higher precursor yield is constrained also by the stoichiometry of the network.

• The dual price of a precursor cannot indicate if this precursor is indeed among the most significant for growth, because the dual price might be related with other needs of the cell. In this case, the indicator is the scaled dual price σ of biomass precursors and of NADPH/NADH, which is estimated as follows [37]:

σ=(Msubstrate)×(∂X∂M)(Xsubstrate).
 MathType@MTEF@5@5@+=feaafiart1ev1aaatCvAUfKttLearuWrP9MDH5MBPbIqV92AaeXatLxBI9gBaebbnrfifHhDYfgasaacH8akY=wiFfYdH8Gipec8Eeeu0xXdbba9frFj0=OqFfea0dXdd9vqai=hGuQ8kuc9pgc9s8qqaq=dirpe0xb9q8qiLsFr0=vr0=vr0dc8meaabaqaciaacaGaaeqabaqabeGadaaakeaaiiGacqWFdpWCcqGH9aqpdaWcaaqaamaabmaabaWaaSaaaeaacqqGnbqtaeaacqqGZbWCcqqG1bqDcqqGIbGycqqGZbWCcqqG0baDcqqGYbGCcqqGHbqycqqG0baDcqqGLbqzaaaacaGLOaGaayzkaaGaey41aq7aaeWaaeaadaWcaaqaaiabgkGi2kabbIfaybqaaiabgkGi2kabb2eanbaaaiaawIcacaGLPaaaaeaadaqadaqaamaalaaabaGaeeiwaGfabaGaee4CamNaeeyDauNaeeOyaiMaee4CamNaeeiDaqNaeeOCaiNaeeyyaeMaeeiDaqNaeeyzaugaaaGaayjkaiaawMcaaaaacqGGUaGlaaa@57C0@

where:

Msubstrate
 MathType@MTEF@5@5@+=feaafiart1ev1aaatCvAUfKttLearuWrP9MDH5MBPbIqV92AaeXatLxBI9gBaebbnrfifHhDYfgasaacH8akY=wiFfYdH8Gipec8Eeeu0xXdbba9frFj0=OqFfea0dXdd9vqai=hGuQ8kuc9pgc9s8qqaq=dirpe0xb9q8qiLsFr0=vr0=vr0dc8meaabaqaciaacaGaaeqabaqabeGadaaakeaadaWcaaqaaiabb2eanbqaaiabbohaZjabbwha1jabbkgaIjabbohaZjabbsha0jabbkhaYjabbggaHjabbsha0jabbwgaLbaaaaa@3A56@ the maximum yield of the precursor (solution of the corresponding L.P.(1))

∂X∂M
 MathType@MTEF@5@5@+=feaafiart1ev1aaatCvAUfKttLearuWrP9MDH5MBPbIqV92AaeXatLxBI9gBaebbnrfifHhDYfgasaacH8akY=wiFfYdH8Gipec8Eeeu0xXdbba9frFj0=OqFfea0dXdd9vqai=hGuQ8kuc9pgc9s8qqaq=dirpe0xb9q8qiLsFr0=vr0=vr0dc8meaabaqaciaacaGaaeqabaqabeGadaaakeaadaWcaaqaaiabgkGi2kabbIfaybqaaiabgkGi2kabb2eanbaaaaa@31E0@ the dual price of the precursor in the solution of the L.P for the maximization of the cellular growth rate [L.P.(2)].

Xsubstrate
 MathType@MTEF@5@5@+=feaafiart1ev1aaatCvAUfKttLearuWrP9MDH5MBPbIqV92AaeXatLxBI9gBaebbnrfifHhDYfgasaacH8akY=wiFfYdH8Gipec8Eeeu0xXdbba9frFj0=OqFfea0dXdd9vqai=hGuQ8kuc9pgc9s8qqaq=dirpe0xb9q8qiLsFr0=vr0=vr0dc8meaabaqaciaacaGaaeqabaqabeGadaaakeaadaWcaaqaaiabbIfaybqaaiabbohaZjabbwha1jabbkgaIjabbohaZjabbsha0jabbkhaYjabbggaHjabbsha0jabbwgaLbaaaaa@3A6C@ the maximum cellular growth rate, i.e. the solution of [L.P.(2)]

The closer to unity a dual price σ is the closer to its maximum yield is the metabolite produced when the cell aims at achieving maximum growth.

## Authors' contributions

ICT reconstructed along with MIK the metabolic network, applied LP analysis for all examined cases and drafted the manuscript. MNK provided his valuable expertise in the *in vivo *physiology of *Z. mobilis *cell cultures along with his knowledge in ethanol production from plant biomass. MIK conceptualized the described work, participated in the reconstruction of the metabolic network, supervised the application of LP analysis by ICT, coordinated the work of all authors towards completion, helped significantly in drafting and finalized the manuscript. All authors have read and accepted the manuscript.

## Appendix 1A

The reconstructed metabolic network of the engineered *Z. mobilis *(C25). All network reactions are depicted in the following format: ***[Reaction Number]. [Reaction Formula]. [(I) or (R), I: if the reaction is considered irreversible or reversible, respectively]. [EC Number of the enzyme that catalyzes the particular reaction]. [Name of the gene that encodes the enzyme that catalyzes the particular reaction]. [Main literature source from which the provided information has been recovered: ***1:[43],2:[22], 3:[21]. All stoichiometric coefficients refer to mole requirements; only the coefficient of biomass refers to mass (g) requirements.

### Entner-Doudoroff (E.D) pathway

1. Glc + ATP → G6P + ADP (I) E.C: 2.7.1.2 (glc) (1)

2. G6P + NADP → glucono1,5lactone6P + NADPH (I) E.C:1.1.1.49 (zwf) (1)

3. Glucono1,5lactone6P + H2O → 6PG (I) E.C:3.1.1.31 (pgl) (1)

4. 6PG → 2K3D6P + H2O (I)E.C:4.2.1.12 (edd) (1)

5. 2K3D6P → PYR + GAP (R) E.C:4.1.2.14 (eda) (1)

### Part of Embden-Meyerhof-Parnas pathway (E.M.P)

6.GAP+ NAD^+ ^→ 1,3BPG+ NADH (R) E.C:1.2.1.12 (gap) (1)

7. 1,3BPG+ADP → G3P+ATP (R) EC: 2.7.2.3 (pgk) (1)

8. G3P → 2PG (R) E.C:5.4.2.1 (pgm) (1)

9. 2PG → PEP+ H2O (R) E.C:4.2.1.11 (eno) (1)

10. PEP + ADP → PYR + ATP (I) E.C:2.7.1.40 (pyk) (1)

11. G6P → F6P (R) E.C.: 5.3.1.9 (pgi) (1)

### Xylose Catabolism

12. Xylose → Xylulose (I) E.C: 5.3.1.5 (xyla)

13. Xylulose + ATP → XYLU5P+ADP (R) E.C 2.7.1.17 (xk)

### Pentose phosphate pathway

14. 6PG +NADP^+ ^→ RIBU5P+CO2+NADPH (I) E.C:1.1.1.44 (gnd) (2)

15. RIBU5P → RI5P (R) E.C:5.3.1.6 (pria) (1)

16. RIBU5P → XYLU5P (R) E.C:5.1.3.1 (rpe) (1)

17. RI5P+ XYLU5P → SED7P(21)+GAP (R) E.C:2.2.1.1 (tklb) (1)

18. SED7P+ GAP → E4P + F6P (R) E.C:2.2.1.2 (tal) (2)

19. XYLU5P + E4P → GAP+F6P (R) E.C: E.C:2.2.1.1 (tklb) (1)

### Fermentation Pathways/Pyruvate Metabolism

20. PYR → AcAld + CO2 (I) E.C.:4.1.1.1 (dcp) (1)

21. AcAld + NADH → Ethanol + NAD^+ ^(I)E.C.:1.1.1.1 (adha) (1)

22. PYR → AcCoA +CO2 (I) E.C:1.2.4.1,2.3.1.12,1.8.1.4 (pdhB), (pdhC),(lpd) (1)

23. PYR+ NADH → D-Lactate + NAD^+ ^(I)E.C.:1.1.1.28 (ldh) (1)

24. AcAld + H2O + NAD^+ ^→ Acetate +NADH (I) E.C:1.2.1.3 (aldh)(3)

25. CIT → Acetate+ OAA (R)E.C:4.1.3.6 (cite) (1)

26. 2PYR → 2-acetolactate + CO2 (I) E.C:2.2.1.6 (budB) (1)

27. 2-acetolactate → (R)-2-acetoin + CO2 (I)E.C:4.1.1.5 (aldc) (2)

### Incomplete (Reductive) Tricarboxylic Acid Cycle (TCA)

28. AcCoA+OAA+H2O → CIT+CoA (I) E.C.:2.3.3.1 (gltA) (1)

29. CIT → Isocitrate + H2O (R) E.C:4.2.1.3 (aconA) (1)

30. Isocitrate + NADP → AKG + CO2 + NADPH (R)E.C:1.1.1.42 (citC) (1)

31. (S)-malate → fumarate + H2O (R) E.C:4.2.1.2 (fumA) (1)

32. fumarate + FADH2 → succinate + FAD + H2O (R) E.C:1.3.99.1 (sdhC) (1)

33. succinate+ ATP+ CoA → sucCoA + ADP + phosphate (R) E.C:6.2.1.5 (sucD) (1)

### Anaplerotic Reactions

34. PYR + CO2 + NADH → (S)-malate + NAD^+ ^(R)E.C:1.1.1.38 (yqkJ) (1)

35. PEP + H2O + CO2 → OAA +Pi (I) E.C:4.1.1.49 (ppc) (1)

### Synthesis of Other Products

36. GAP → GlyceroneP (R) E.C:5.3.1.1 (tpi) (1)

37. GLP + NADH → sn-GL3P + NAD (R) E.C:1.1.1.94(gpsA) (1)

38.sn-GL3P + H2O → Glycerol + Pi (R) E.C. 3.1.3.21 (1, based on deGraff)

39. Xylulose + NADH → Xylitol + NAD (I)EC:1.1.1.9 (xyld) (2)

40. PYR + CoA → AcCoA + formate (I)E.C:2.3.1.54 (pfl) (1)

### Elecron transfer system and dehydrogenases

41. NADH + Q → NAD + QH2 + 2H^+ ^(I) E.C:1.6.5.3 (ndh1) (1)

42. NADH + Q → NAD + QH2 (I) E.C:1.6.99.3. (ndh2) (1)

43. FADH2 +Q → FAD +QH2 + 2H^+ ^(R) (3)

44. QH2 + 2 ferricytocrome c → Q + 2 ferrocytochrome c + 2H^+ ^(I)

E.C:1.10.2.2 (uqr) (3)

45. Formate + Q → CO2 + QH2 + 2H^+ ^(I) (1.2.2.1 in combination with reaction 40)

46. ADP + Pi + 3H^+ ^→ ATP (I) E.C.:3.6.3.14,3.6.1.1,2.7.4.1 (atpase) (3)

47. NADPH → NADH (R)

### Transport reactions

48. Glucose Ext → Glucose (I)

49. Xylose Ext → Xylose (I)

50.*CO*_2 _→ *CO*_2 _Ext (R)

51. ATP → ATP Ext (I)

52. AcAld → AcAld Ext (I)

53. succinate → succinate Ext (I)

54. Ethanol → Ethanol Ext (I)

55. D-Lactate → Lactate Ext (I)

56. Acetate → Acetate Ext (I)

57. (R)-2-acetoin → acetoin Ext (I)

58. Glycerol → Glycerol Ext (I)

59. Xylitol → Xylitol Ext (I)

60. Formate → Formate Ext(I)

### Biomass Synthesis

61. PYR - CO2 + NADPH → Ala

62. AKG + CO2 + 4NADPH → Arg

63. OAA + NADPH → Asx

64. G3P + 5NADPH - NADH → Cys

65. AKG + NADPH → Gln

66. G3P + NADPH -NADH → Gly

67. RI5P + NADPH -2NADH → His

68. PYR + OAA + 5NADPH → Ile + CO2

69. 2PYR + AcCoA + 2NADPH - NADH → Leu + 2CO2

70. Pyr + OAA + SUCCoA + 4NADPH → Lys + CO2 + Suc + CoA

71. OAA + 8NADPH - NADH → Met

72. E4P+ 2PEP + 2NADPH → Phe + CO2

73. AKG + 3NADPH → Pro

74. G3P + NADPH - NADH → Ser

75. OAA + 3NADPH → Thr

76. RI5P + E4P + PEP + 2NADPH - NADH → Trp + CO2

77. E4P + 2PEP + 2NADPH - NADH → Tyr + CO2

78. 2PYR + 2NADPH → Val + CO2

79. 0.000154G6P +0.00019F6P +0.000823RI5P +0.00012GAP +0.001705G3P +0.000311PEP +0.03867PYR +0.002607AcCoA +0.002878OAA +0.000829AKG -0.001344CO2 +0.017202NADPH +0.000829AKG +0.000249SuccinylCoA -0.000249Succinate -0.00252NADH +0.001088Ala +0.000181Arg +0.000478Asx +0.00002Cys +0.000343Glx +0.00092Gly +0.000082His +0.000369Ile +0.000369Leu +0.000249Lys +0.000081Met +0.000011Phe +0.00021Pro +0.000202Ser +0.000224Thr +0.000054Trp +0.00007Tyr +0.000569Val = 1g Biomass

## Appendix 1B

List of the network metabolites and their utilized abbreviations

1. Glucose *Glc*

2. Adenosine triphosphate *ATP*

3. Glucose-6-phosphate *G6P*

4. D-glucono-1,5-lactone6-phosphate glucono1,5lactone6P

5. Nicotinamide Adenine Dinucleotide Phosphate (reduced) *NADPH*

6. 6-phosphogluconolactonase *6PG*

7. 2-dehydro-3-deoxy-6-phospho-D-gluconate *2K3D6P*

8. Pyruvate *PYR*

9. D-glyceraldehyde3-phosphate *GAP*

10. 3-phospho-D-glyceroylphosphate *1,3BPG*

11. Nicotinamide adenine dinucleotide (reduced) *NADH*

12. 3-phospho-D-glycerate *G3P*

13. 2-phospho-D-glycerate *2PG*

14. Phosphoenolpyruvate *PEP*

15. Fructose 6-phosphate *F6P*

16. Xylose

17. Xylulose

18. Xylulose5-phosphate *XYLU5P*

19. Carbon dioxine *CO*_2_

20. Ribulose-5-phosphate *RIBU5P*

21. Ribose-5-phosphate *RI5P*

22. Sedoheptulose7-phosphate *SED7P*

23. Erythrose4-phosphate *E4P*

24. Acetaldehyde *AcAld*

25. Ethanol

26. Acetyl coenzyme A *AcCoA*

27. Lactate

28. Acetate

29. Citrate *Cit*

30. Oxaloacetate *OAA*

31. 2-acetolactate

32. (R)-2-acetoin

33. Isocitrate

34. α-Ketoglutarate *aKG*

35. (S)-malate

36. Fumarate

37. Flavin Adenine Δinucleotide *FADH*_2_

38. Succinate *Suc*

39. Succinyl CoA *SucCoA*

40. Glycerone-phosphate *Glycerone-P*

41. Sn-Glycerol3-phosphate *GL3P*

42. Glycerol

43. Xylitol

44. Formate

45. Ubiquinol *QH2*

46. Protons translocating membrane *H*^+^

47. Glucose Externally Excreted *Glc Ext*

48. Xylose Externally Excreted *Xylose Ext*

49. ATP Externally Excreted *ATP Ext*

50. CO_2 _Externally Excreted *CO*_2_*Ext*

51. AcAld Externally Excreted *AcAld Ext*

52. Succinate Externally Excreted *Suc Ext*

53. Ethanol Externally Excreted *Ethanol Ext*

54. Lactate Externally Excreted *Lactate Ext*

55. Acetate Externally Excreted *Acetate Ext*

56. Acetoin Externally Excreted *Acetoin Ext*

57. Glycerol Externally Excreted *Gycerol Ext*

58. Xylitol Externally Excreted *Xylitol Ext*

59. Formate Externally Excreted *Formate Ext*

60. Alanine *Ala*

61. Arginine *Arg*

62. Aspartate Asx

63. Cysteine *Cys*

64. Glutamine *Gln*

65. Glycine *Gly*

66. Histidine *His*

67. Isoleucine *Ile*

68. Leucine *Leu*

69. Lysine *Lys*

70. Methionine *Met*

71. Phenylanine *Phe*

72. Proline *Pro*

73. Serine *Ser*

74. Threonine *Thr*

75. Tryptophan *Trp*

76. Tyrosine *Tyr*

77. Valine *Val*

## Appendix 1C

### Linear Programming for the Analysis of Metabolic Networks

For a metabolic network of M metabolites and N metabolic reactions, which is at metabolic steady-state conditions (i.e. all reaction fluxes, including the growth rate, are constant), the linear programming problem is defined as follows [36]:

max⁡z=∑j=1Ncjvj
 MathType@MTEF@5@5@+=feaafiart1ev1aaatCvAUfKttLearuWrP9MDH5MBPbIqV92AaeXatLxBI9gBaebbnrfifHhDYfgasaacH8akY=wiFfYdH8Gipec8Eeeu0xXdbba9frFj0=OqFfea0dXdd9vqai=hGuQ8kuc9pgc9s8qqaq=dirpe0xb9q8qiLsFr0=vr0=vr0dc8meaabaqaciaacaGaaeqabaqabeGadaaakeaafaqabeqacaaabaGagiyBa0MaeiyyaeMaeiiEaGhabaGaemOEaONaeyypa0ZaaabCaeaacqWGJbWydaWgaaWcbaGaemOAaOgabeaakiabdAha2naaBaaaleaacqWGQbGAaeqaaaqaaiabdQgaQjabg2da9iabigdaXaqaaiabd6eaobqdcqGHris5aaaaaaa@3FF1@

***subject to***:

S¯¯⋅v¯=(r¯output−r¯input)
MathType@MTEF@5@5@+=feaafiart1ev1aaatCvAUfKttLearuWrP9MDH5MBPbIqV92AaeXatLxBI9gBaebbnrfifHhDYfgasaacH8akY=wiFfYdH8Gipec8Eeeu0xXdbba9frFj0=OqFfea0dXdd9vqai=hGuQ8kuc9pgc9s8qqaq=dirpe0xb9q8qiLsFr0=vr0=vr0dc8meaabaqaciaacaGaaeqabaqabeGadaaakeaadaadbaqaaiabdofatbaacqGHflY1daadaaqaaiabdAha2baacqGH9aqpcqGGOaakdaadaaqaaiabdkhaYbaadaahaaWcbeqaaiabd+gaVjabdwha1jabdsha0jabdchaWjabdwha1jabdsha0baakiabgkHiTmaamaaabaGaemOCaihaamaaCaaaleqabaGaemyAaKMaemOBa4MaemiCaaNaemyDauNaemiDaqhaaOGaeiykaKcaaa@486D@ (*metabolite balance constraints*)

x¯≤v¯≤y¯
 MathType@MTEF@5@5@+=feaafiart1ev1aaatCvAUfKttLearuWrP9MDH5MBPbIqV92AaeXatLxBI9gBaebbnrfifHhDYfgasaacH8akY=wiFfYdH8Gipec8Eeeu0xXdbba9frFj0=OqFfea0dXdd9vqai=hGuQ8kuc9pgc9s8qqaq=dirpe0xb9q8qiLsFr0=vr0=vr0dc8meaabaqaciaacaGaaeqabaqabeGadaaakeaadaadaaqaaiabdIha4baacqGHKjYOdaadaaqaaiabdAha2baacqGHKjYOdaadaaqaaiabdMha5baaaaa@34AF@ (*constraints on the lower and upper bound for flux values*)

where *z*, *c*_*j *_depict, respectively, the cellular objective as linear function of the flux vector and the weight of the j-th flux in this linear function

In this problem, the feasible values of the reaction fluxes (or in LP terms, the feasibility space of the flux vector) are constrained by (a) the stoichiometry of the (maximum potentially active) network, as this is imposed through the metabolite balance constraints, and (b) lower and upper bounds, which are determined from previous biological knowledge (if no special bounds are to be imposed on a particular flux, x and y are -8 and +8, respectively). Since the maximum potentially active network depends on which enzymes are producible from the particular organism, thus on which genes encoding for these enzymes exist in this organism's genome, the stoichiometrically feasible flux space has been termed "metabolic genotype" [52]. The *in vivo *metabolic flux distribution is a point of this space. If nonlinear regulatory mechanisms, which are active in a metabolic network, are also considered, the feasible domain for the metabolic flux values will be a subset of the stoichiometrically feasible. This is the reason behind the argument that linear programming analysis is the first level of metabolic network analysis. It seeks to identify the boundaries of the network in achieving particular (linear) objective(s), according to its stoichiometry only. If the LP problem does not have any solution, this means that the stoichiometry of the network prevents it from reaching this particular objective. In this case, the network cannot achieve the particular objective under any circumstances. However, if the LP does have a solution, this does not necessarily mean that the cell can indeed achieve a particular objective. The metabolism of the cell is under the control of local and global regulatory mechanisms that may prevent the realization of a particular physiological state. Since these regulatory mechanisms are not taken into consideration in LP analysis, *in vivo *data are needed to enhance the linear stoichiometric model to one including regulation, which is closer to *in vivo *reality.

In the case of gene deletions, the stoichiometrically feasible flux domain changes. The gene deletion analysis (which is also presented in this study for single and double gene deletions) provides insight to how crucial a particular reaction/flux might be for the realization of a cellular objective. In addition, through the "shadow or dual" prices, LP analysis provides information about the effect that a change in a particular metabolite's net excretion rate could have on the value of the objective function. For all these reasons, LP analysis is considered necessary first level of metabolic network analysis for obtaining information about the interconnectivity of the stoichiometric model [52].
